# Correlation versus causation: *Helicobacter pylori* population heterogeneity complicates the identification of mutant strain phenotypes

**DOI:** 10.1128/msphere.00638-25

**Published:** 2025-12-15

**Authors:** Marina R. Wylie, Jeremy J. Gilbreath, Angela Melton-Celsa, D. Scott Merrell

**Affiliations:** 1 Department of Microbiology and Immunology, Uniformed Services University of the Health Sciences 1685, Bethesda, Maryland, USA; 2 Henry M. Jackson Foundation for the Advancement of Military Medicine, Inc. 44069 , Bethesda, Maryland, USA; University of Michigan, Ann Arbor, Michigan, USA

**Keywords:** *H. pylori*, heterogeneity, biofilm, motility, *sabA*

## Abstract

*Helicobacter pylori* causes cancer in approximately 1% of infected individuals. A proposed mechanism of *H. pylori* persistence centers on the ability of the pathogen to form biofilms, yet little is known about specific genetic requirements for this process. Our investigation revealed that during lab passage, *H. pylori* accumulates genetic changes that impact further phenotypic analyses. Specifically, we first sought to characterize the roles of the flagellar genes, *pflA* and *flgS*, in biofilm formation; the *flgS* mutant strain was biofilm deficient, but the *pflA* mutant strain was a hyper-biofilm former; however, the *pflA* mutant strain phenotype was unstable. Analysis and screening of six new *pflA* mutant strains revealed variable biomass phenotypes. This unexpected result led us to explore how genetic heterogeneity within an *H. pylori* population may complicate standard mutagenesis processes and the interpretation of downstream phenotypes. Analysis of single colony isolates from multiple wild-type strains similarly yielded different biomass phenotypes despite the expected isogenic nature of these isolates. Genomic sequencing of a subset of these isolates revealed various nucleotide changes. Analysis of some of these changes revealed that mutation of *futB*, *babA*, or *babB* did not affect biofilm formation, while mutation of *sabA*, which encodes the SabA adhesion, resulted in a significant decrease in *H. pylori* biofilm formation. Overall, these findings reveal that nucleotide changes that occur during a single passage of *H. pylori* may impact downstream phenotypic analyses. Moreover, these data emphasize the necessity of genetic confirmation redundancy and/or complementation to conclusively move from correlation to causation when analyzing phenotypes of constructed mutant strains.

**IMPORTANCE:**

*H. pylori* displays a high rate of genetic variability, but many studies still do not utilize independent confirmation or complementation to conclusively demonstrate that mutations of interest are responsible for identified phenotypes. Our attempts to study biofilm formation were stymied by the realization that individual colonies cultured from wild-type strains had numerous genetic changes despite their expected isogenic nature; these changes correlated with phenotypic differences for individual wild-type isolates. Analysis of a subset of these genetic changes revealed that correlation and causation were not always linked. However, constructed mutations and natural variation in *sabA* both dramatically decreased biofilm formation. Overall, the extensive genetic heterogeneity that exists within individual cells within an *H. pylori* population may affect phenotypes of interest; this serves to emphasize the necessity of redundant methods of strain construction, sequence confirmation, and/or genetic complementation to conclusively move from correlation to causation for any phenotype of interest.

## INTRODUCTION

*Helicobacter pylori* successfully colonizes approximately 50% of the world’s population (1). Gastritis and gastric ulcers are common in those who are infected with *H. pylori*, and a subset of the infected population will develop severe disease (i.e., gastric cancer) (2). Given the wide range of possible severe disease outcomes, a significant amount of *H. pylori* research has focused on the identification of genetic components and virulence factors that are key determinants of pathogenesis. For example, the *H. pylori* oncoprotein, cytotoxin, and outer membrane proteins encoded by *cagA*, *vacA*, *homB*, and *sabA*, respectively, have been well characterized and are epidemiologically and/or genetically associated with severe disease and/or contribute to disease progression in animal models (3–9). In addition to these classical virulence factors, more recent studies have identified biofilm formation as a potential mechanism of *H. pylori* persistence and pathogenesis (10–14).

Biofilm formation is employed by many different pathogens, particularly by those that cause chronic infections (15, 16). These bacterial communities provide protection from outside stressors, including those from the immune system, and often increase antibiotic tolerance, which can contribute to persistence and treatment failure (11, 17–22). Several groups have begun to characterize *H. pylori* biofilms *in vitro*; most of those studies utilized “-omics” techniques, tested various treatments, and/or focused on a single gene or system of interest (10, 23–32). So far, flagellar genes, specific outer membrane proteins, and the acid-responsive two-component system encoded by *arsRS* are a few of the factors with demonstrated roles in *H. pylori* biofilm formation (24, 33–37). However, *H. pylori* biomass production and extracellular matrix composition *in vitro* varys greatly depending on the assay, surface, medium, strain, and time point being studied (11, 38). Based on this variation and the limited understanding of the role of any factors in *in vivo* biofilm formation (12, 13, 39, 40), more insight into the role of *H. pylori* biofilm formation as a potential pathogenic mechanism is needed.

As a species, *H. pylori* is known to display a great deal of variability across strains (41–46). Moreover, *H. pylori* is known to employ a wide range of genetic mechanisms of variation (47, 48). Our current understanding suggests that a genotypically and phenotypically diverse population can be beneficial to a chronic colonizer such as *H. pylori*; this pathogen must establish a niche in the inhospitable stomach environment and then evade host defenses for decades (49). In line with this idea, it is known that *H. pylori* undergoes an *in vivo* mutational burst soon after infection, which leads to genetically unique strains from colonized individuals; this is supported by clinical isolate diversity and by experimental infections of non-human primates (50–58). Some of the well-studied features of the *H. pylori* genome and mechanisms for genetic variation include natural competence, an abundance of polynucleotide tracts that facilitate slipped-strand mispairing and phase variation, frequent recombination, an error-prone DNA polymerase, and a lack of mismatch repair system components (44, 47, 59, 60). These characteristics lead to an overall mutation rate that is much higher than other bacterial species both *in vivo* and *in vitro*; in some experiments, this rate is 100-fold greater for *H. pylori* and up to 10^−4^ changes per site per year during the acute phase of infection (50, 59, 61, 62). Although this knowledge is widely accepted in the *H. pylori* field, the methods used to test and screen for phenotypes *in vitro* have not always accounted for the high levels of genetic heterogeneity that exist within a population of *H. pylori* cells. In particular, the selection and expansion of single colonies during the construction of isogenic mutant strains, transposon library screens, and other forms of genetic manipulation might lead to incomplete or misleading results; genetic changes may occur within a single passage that affect complex phenotypes of interest. A publication by Draper et al. sought to address the “Fallacy of the Unique Genome” and thoroughly compared genomic sequences of “working stock” populations of *H. pylori* SS1 isolates before and after mouse passage (63). Given the *in vivo* selection pressures imposed by murine passage, many mutations were identified, some with implications for virulence and colonization factor expression; however, these two genomes were still 99.9% identical. Using SS1 strains as a model, Draper et al. concluded that (i) consensus genome assemblies from single colonies could be misleading and might not represent the variability present and (ii) high-depth population-level genomic sequencing data can provide insight into “normal” variation within bacterial strains (63). Though less well understood, it is possible that *in vitro* laboratory passage, genetic manipulation, and screening methods could also be affected by *H. pylori* population heterogeneity and genetic plasticity; therefore, additional practical studies are likely needed to assess these potential effects and to determine how they might complicate downstream assay interpretation.

Here, we describe how, during our attempts to characterize genes that have roles in biofilm formation and motility, we identified phenotypic differences within presumably isogenic strains. This finding led us to study the inherent genetic variability of wild-type (WT) *H. pylori* strains and to determine how this heterogeneity can, in practice, affect the investigation of complex phenotypes such as biofilm formation and motility. We found that within a single passage, multiple *H. pylori* WT and mutant strain backgrounds showed evidence of high mutation rates, significant biofilm formation and motility variability, and frequent loss of identified phenotypes upon further *in vitro* passage. Whole-genome sequencing (WGS) of single colony isolates from WT *H. pylori* G27 and SS1 strains revealed many nucleotide changes that correlated with identified biofilm phenotypes. However, further characterization of a subset of these showed that many of these changes did not seem to be responsible for the identified phenotypes. Despite this challenge, a role for the outer membrane protein-encoding gene, *sabA*, in *H. pylori* biofilm formation was identified using several approaches. Overall, our findings emphasize the necessity of redundant methods of strain construction, sequence confirmation, and/or genetic complementation to conclusively move from correlation to causation for any phenotype of interest.

## RESULTS

To begin to investigate the contributions of the flagellar genes *flgS* and *pflA* to *in vitro* biofilm formation by *H. pylori* G27, individual deletion mutant strains were constructed and tested for motility and biomass phenotypes in comparison to the WT parent strain. FlgS is the histidine kinase of the FlgRS two-component system that regulates the transcription of flagellar genes (64). PflA, or paralyzed flagellar protein A, has been identified as an integral part of the flagellar motor in *Campylobacter*, *H. pylori*, and other related species (65, 66). Mutation of *flgS* results in cells that are non-flagellated and non-motile, whereas *pflA* mutation results in cells that are flagellated but non-motile due to paralyzed flagella (23, 67, 68). As expected, both G27 Δ*flgS::cat* and G27 Δ*pflA::cat* were non-motile in a soft agar assay (data not shown). Crystal violet staining of 72 h biofilms revealed that, consistent with previously published data (23), the G27 Δ*flgS::cat* mutant strain was biofilm deficient and served as an effective control (Fig. S1A). However, the G27 Δ*pflA::cat* strain displayed a significantly higher level of biomass as compared to WT (Fig. S1A). The biofilm CFU data followed the same patterns, whereas no significant differences in planktonic CFU were detected for either of the mutant strains (Fig. S1B and C, respectively). In line with previous findings by Hathroubi et al., these results suggest that motility *per se* is not required for biofilm formation in *H. pylori* G27, but flagella may instead play a key structural role in biofilm formation by this species (23).

To further investigate the roles of *pflA* and paralyzed flagella in *H. pylori* biofilms, the freezer stock of the original G27 Δ*pflA::cat* mutant strain (DSM709) was expanded with antibiotic selection to ensure maintenance of the *cat* cassette and then archived. However, when the new stock was later tested in the biofilm assay, the hyper-biofilm phenotype of the original Δ*pflA::cat* mutant strain was no longer detected (Fig. S1D); planktonic CFU was also comparable (Fig. S1F), and PCR using genomic DNA (gDNA) isolated from the new stock confirmed that the Δ*pflA::cat* mutation was still present in the strain (data not shown). This finding suggested that second-site mutations/nucleotide changes within the genome of the Δ*pflA::cat* mutant were convoluting the interpretation of the originally observed biofilm phenotype.

### Isogenic mutant strains display variable biofilm phenotypes and accumulate an abundance of secondary mutations

**Fig 1:**
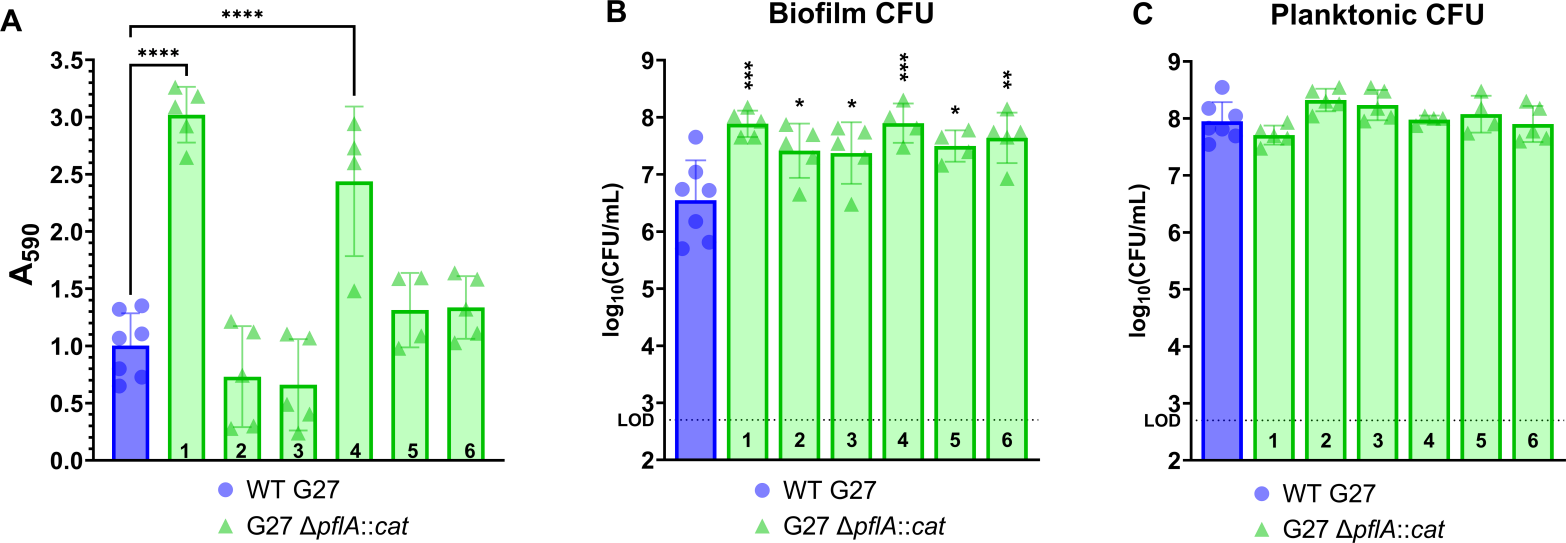
Six isogenic mutant strains display variable biofilm phenotypes at 72 h. (**A**) OD-controlled overnight liquid cultures were adjusted to an OD_600_ of 0.1 and added to a 24-well plate. After 72 h, the biofilms were washed with brucella broth (BB), allowed to dry, and then stained with 1% crystal violet. Stained biomass was resuspended in an alcohol solution, and the absorbance was read at 590 nm. (**B and C**) A duplicate set of wells was used to collect planktonic and biofilm CFU for enumeration. CFU data were log-transformed before analysis; the limit of detection (LOD, dotted line) was 500 CFU. Numbers (1–6) indicate individual isolates of the indicated genotype. One-way ANOVAs with Dunnett correction were performed; *n* ≥ 4; individual data points are plotted with the mean and SD; ****P < 0.0001, ***P ≤ 0.0007, **P = 0.0035, and *P < 0.04 as compared to WT G27.

To investigate the observed loss of the original G27 Δ*pflA::cat* hyper-biofilm phenotype upon expansion, the *pflA* mutant strain was reconstructed. To this end, the Δ*pflA::cat* locus from DSM709 gDNA was PCR amplified, cleaned, and transformed into DSM1 (WT G27). Six individual colonies from a single transformation were chosen and expanded with antibiotic selection to create freezer stocks; all six mutant strain isolates were confirmed to contain the expected mutation via PCR and to be non-motile in the soft agar motility assay (data not shown). These six isolates were then tested in the 72 h biofilm assay. Resultant biofilm phenotypes could be broadly grouped into three categories; two of the six new G27 Δ*pflA::cat* mutant strains produced significantly more biomass than the WT parent strain, two of the six produced slightly less biomass than WT, and two produced biomass that was similar to, but slightly higher than, WT (Fig. 1A). Only isolates #1 and #4 were significantly different from WT G27. In contrast, all six of the new G27 Δ*pflA::cat* mutant strains showed significantly higher levels of biofilm CFU in comparison to WT G27, indicating a degree of maintenance of the original hyper-biofilm phenotype when assessed by this metric (Fig. 1B). Similar to the aforementioned biofilm experiments (Fig. S1C and F), planktonic CFU from all six new mutant strains were not significantly different from WT G27 (Fig. 1C).

The observed variable biomass levels displayed by these supposed “isogenic” mutant strains led us to investigate background genetic heterogeneity and the occurrence of secondary mutations in the G27 Δ*pflA::cat* mutant strain isolates. To this end, G27 Δ*pflA::cat* #1, #3, and #6 were selected for WGS and variant calling analysis (69); these three isolates were selected because they each represented one of the three categories of identified biomass phenotypes (Fig. 1A). To identify any sequence differences from the published G27 genome (GenBank CP001173.1) (70), the WT G27 parent strain (DSM1) was also sequenced and analyzed.

Comparison of the highly passaged Merrell lab WT G27 strain (DSM1) to the published G27 genome revealed a total of 56 nucleotide differences that were present with at least 80% frequency within the sequencing reads. These nucleotide differences were distributed across the genome and spanned both coding sequences (CDSs) (23 total) and intergenic regions (33 total). Twenty-eight of the identified changes occurred within polynucleotide tracts, six of which were within CDSs. Also within CDSs, there were eight single-nucleotide polymorphisms (SNPs) that were silent changes, four single-nucleotide insertions predicted to result in frameshifts, three SNPs that resulted in amino acid changes, and two multi-nucleotide substitutions (Table S1). Given that DSM1 has been passaged under laboratory conditions for decades, and because *H. pylori* strains are known to mutate relatively frequently, in part because of an abundance of variable regions and polynucleotide tracts across the genome (44), the identification of nucleotide differences from the published G27 sequence was not unexpected. However, some of these changes occurred within the CDSs of known virulence and colonization factors (e.g., *cagA* and *babB*, respectively). Of note, alteration of these genes could influence *H. pylori* phenotypes such as cytotoxicity and adherence, respectively (71, 72).

To delineate the nucleotide changes that were unique to the Δ*pflA::cat* background (i.e., not present in the parent WT G27 strain) and to correlate these changes with significantly higher biomass (only in Δ*pflA::cat* #1), slightly lower biomass (only in Δ*pflA::cat* #3), or slightly higher levels of biomass (only in Δ*pflA::cat* #6), the genomes of the three Δ*pflA::cat* isolates were compared to the DSM1 genome. Variant calling analyses revealed that the genomes of these three mutant isolates were similar to the WT G27 parent strain, DSM1; Δ*pflA::cat* mutants #1 and #3 lost only one SNP each that was detected in DSM1 (Table S1). In other words, these two loci reverted to the published G27 sequence (CP001173.1); all other changes found identified in DSM1 as compared to the published G27 genome were maintained in the mutant isolates. However, several additional nucleotide changes were identified in the three Δ*pflA::cat* mutant isolates; most of these changes were unique to a specific isolate. For example, 19 nucleotide changes were detected in *babA* in Δ*pflA::cat* mutant #3; 16 of these changes were unique to this isolate, while the others were shared with Δ*pflA::cat* mutant #6 (Table S1). Why *babA* appeared to be a “hot spot” for change in these isolates remains unknown; however, *H. pylori* encodes many different outer membrane proteins, and these changes may indicate recombination with another *bab* or outer membrane protein-encoding gene (73–76). Given the role of BabA as an adhesin, the changes detected in *babA* could affect aggregation and/or adherence of Δ*pflA::cat* mutant #3, which could in turn contribute to the decreased levels of biomass produced by this isolate (77, 78). However, with the abundance and variety of genetic changes detected across the sequenced mutant isolates (Table S1), it was difficult to determine exactly which individual change(s) or combination of SNPs contributed to the biomass level of a given isolate; it is also possible that epistatic and/or compensatory interactions between the observed changes may affect observed phenotypes. Furthermore, it was unclear if the additional genetic changes identified in the Δ*pflA::cat* isolates occurred during normal lab passage or were the result of the mutagenesis process used to construct these strains.

### Single colony isolates from WT strains also display significant phenotypic variation in biomass and motility

**Fig 2:**
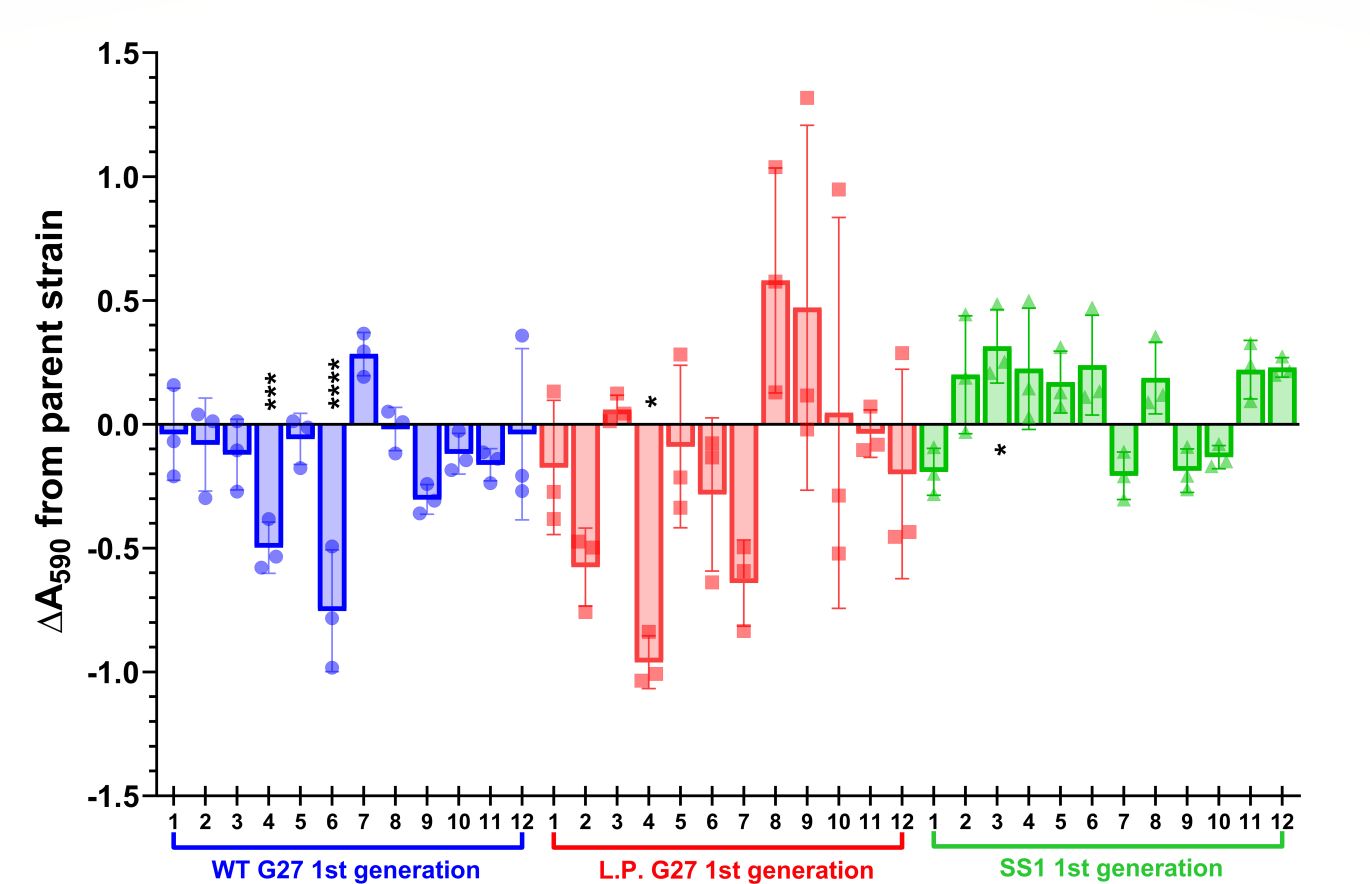
Biofilm formation by lab WT G27 (blue), L.P. G27 (red), and SS1 (green) first-generation single colony isolates. Seventy-two-hour biomass (A_590_) of each first-generation isolate (designated #1–12 for each strain) was normalized to the respective parent strain within each biological replicate. OD-controlled liquid overnight cultures were adjusted to an OD_600_ of 0.1 and added to a 24-well plate. After 72 h, the biofilms were washed with brucella broth (BB), allowed to dry, and then stained with 1% crystal violet. Stained biomass was resuspended in an alcohol solution, and the absorbance was read at 590 nm. Biofilms of SS1 parent strain and first-generation isolates were grown in BB + 1% FBS, while WT G27 and L.P. G27 were grown in BB10. A one-way ANOVA with Dunnett correction was performed on normalized data for each set of 12 isolates; *n* ≥ 3; individual data points are plotted with the mean and SD; ****P < 0.0001, ***P = 0.0001, and *P < 0.05 for the indicated isolate as compared to the respective parent strain.

To determine whether the observed variations in biofilm formation were unique to the G27 Δ*pflA::cat* mutant strains or if similar levels of heterogeneity could be detected among WT isolates as well, a single colony plating and screening approach was next used (Fig. S2). To this end, 36 first-generation single colony isolates were assessed; these isolates represented 12 single colonies each from three parent strains: highly passaged WT G27 (DSM1), low-passage (L.P.) G27 (DSM359), and SS1 (DSM136). We reasoned that comparison of the lab WT G27 to L.P. G27 would identify differences resulting from long-term lab passage of G27, and comparison to SS1 would identify differences specific to the G27 strain lineage. All 36 first-generation isolates were screened for biomass production at 72 h, and the results were visualized as changes in crystal violet staining as compared to the respective parent strain (Fig. 2). Overall, a high level of biofilm variability was observed across these isolates, even between those from the same parent strain; this variability was relatively consistent across multiple biological replicates, regardless of passage (WT G27 compared to L.P. G27) or strain background (G27 strains compared to SS1). Many of the differences were visually striking when plotted, and 4 of 36 isolates displayed statistically significant differences in biomass accumulation as compared to the respective parent strain (~11% of analyzed isolates; Fig. 2). The magnitude of biofilm variation was smaller for SS1-derived isolates, but this may be due to the fact that SS1 isolates generally produced less biomass and contained fewer biofilm CFU than G27 isolates (WT and L.P.) in this assay (Fig. S3); this was true even in conditions optimized for SS1 strains (i.e., media with less fetal bovine serum [FBS] [23]; data not shown). Notably, despite significant biomass results or trends, none of the 36 first-generation single colony isolates displayed significantly different levels of biofilm CFU when compared to the respective parent strain (Fig. 2; Fig. S3A, C and E); however, this result is not uncommon for *H. pylori in vitro* biofilm assays and may suggest that differences in biomass production, not attachment or growth, mainly determine biofilm phenotypes in this assay (27, 38, 79). Planktonic CFU remained consistent across all first-generation single colony isolates as compared to the respective parent strain (Fig. S3B, D and F).

**Fig 3:**
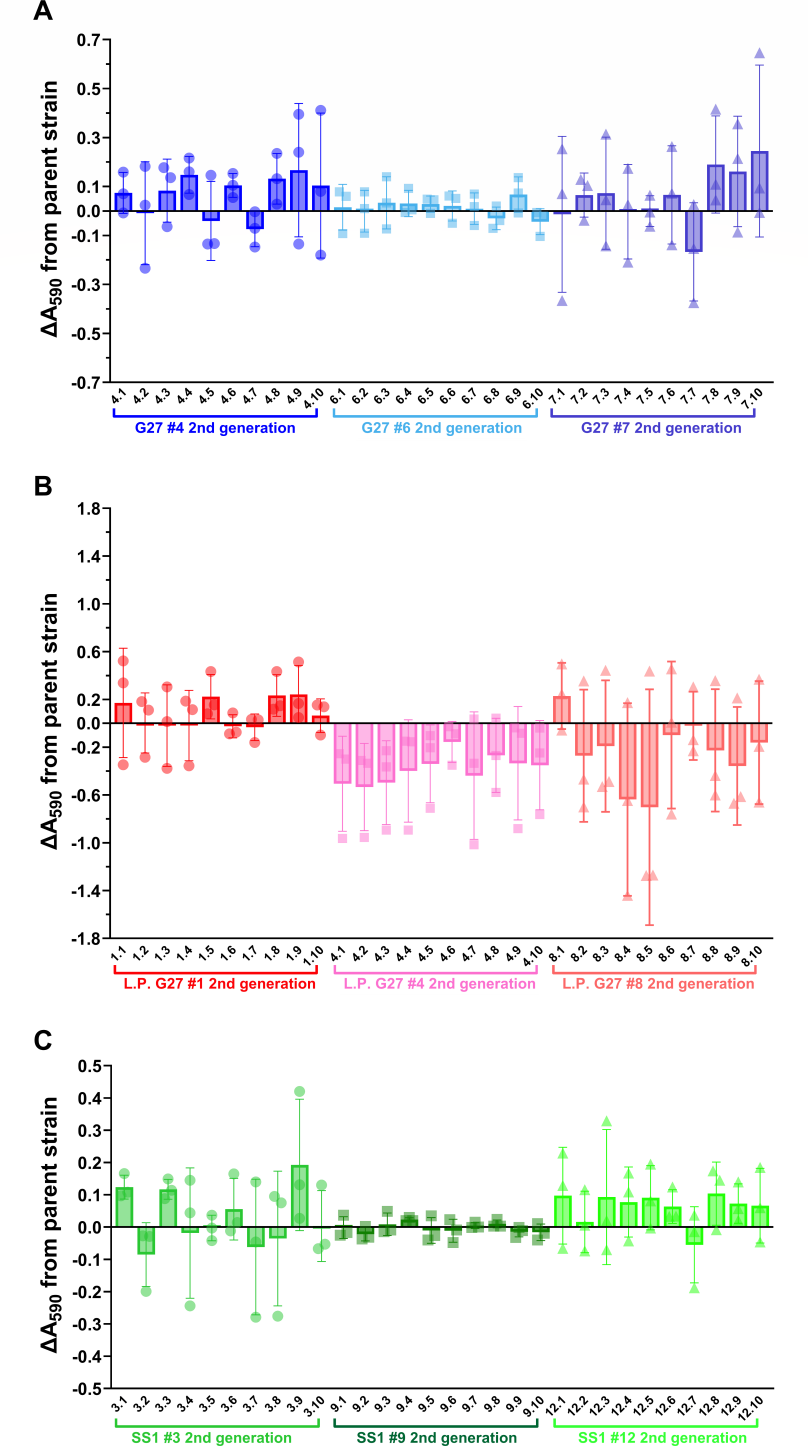
Biofilm formation by second-generation single colony isolates. (**A**) Biomass produced by second-generation isolates derived from lab WT G27 #4, #6, and #7 (blue). (**B**) Biomass produced by second-generation isolates derived from L.P. G27 #1, #4, and #8 (red). The y-axis in panel B is expanded to accommodate the range of biomass differences detected among the L.P. G27 isolates. (**C**) Biomass produced by second-generation isolates derived from SS1 #3, #9, and #12 (green). OD-controlled overnight liquid cultures were adjusted to an OD_600_ of 0.1 and added to a 24-well plate. After 72 h, the biofilms were washed with brucella broth (BB), allowed to dry, and then stained with 1% crystal violet. Stained biomass was resuspended in an alcohol solution, and the absorbance was read at 590 nm. Biofilms of SS1 isolates were grown in BB + 1% FBS, while WT G27 and L.P. G27 were grown in BB10. Seventy-two-hour biomass (A_590_) of each second-generation isolate was normalized to the respective first-generation parent strain within each biological replicate. A one-way ANOVA with Dunnett correction was performed on normalized ΔA_590_ data for each set of 10 isolates; *n* ≥ 3; individual data points are plotted with the mean and SD; no significant differences were detected as compared to the respective parent strain.

Given the significant phenotypic variation detected among the first-generation single colony isolates, the same plating and screening approach was next used to determine if the observed phenotypes were maintained in second-generation isolates that were derived directly from a subset of the original single colony isolates (Fig. S2). To this end, three first-generation isolates from each of the parent strains were selected for further study; an isolate showing lower, higher, or similar biomass phenotypes as compared to the parent was chosen for each strain (WT G27, L.P. G27, and SS1). These 9 isolates were plated, and 10 individual single colonies from each (90 total) were selected for characterization in the 24-well biofilm assay. Crystal violet staining (A_590_) for each was then plotted relative to the respective first-generation parent isolate (Fig. 3). In most cases, second-generation isolates maintained the biomass phenotypes of the respective parent strain; this was particularly true for lab WT G27 and SS1 isolates, for which the ΔA_590_ values fell within the −0.5 to +0.5 range (Fig. 3A and C, respectively). Furthermore, there were no statistically significant biofilm CFU differences among any of the WT G27 or SS1 second-generation isolates (Fig. S4 and S6). However, L.P. G27 isolates showed more variability overall; many of these second-generation isolates displayed biomass levels that trended away from the parent first-generation isolate (Fig. 3B). For example, L.P. G27 #4.1–4.10 was even further decreased in biofilm formation than the first-generation parent strain, #4. In contrast, the biomass levels for L.P. #8.2–8.10, whose parent, #8, displayed increased biofilm formation, trended back toward the biomass level of L.P. G27. Some of the second-generation isolates from L.P. G27 #8 showed significantly reduced biofilm CFU, even though biomass differences between replicates did not reach statistical significance (Fig. S5E; Fig. 3B). No other CFU trends were detected among the 90 total second-generation single colony isolates (Fig. S4 to S6). Thus, second-generation isolate biofilm phenotypes tended to be stable in the strains that had been lab passaged the longest (lab WT G27 and SS1). Taken together, these data may suggest that the L.P. G27 strain, which has been minimally passaged as compared to the lab WT G27 and SS1 strains, may be accumulating genetic changes at a higher rate because of the minimal lab passage of the strain. This finding would be in keeping with studies in other bacterial systems that have shown the tendency of lab-passaged strains to adapt to the lab environment via the accumulation of genetic changes (44, 80–82).

To determine whether the high level of biomass variability that was observed across the isolates was specific to that phenotype, we next assessed the 36 first-generation isolates and 90 second-generation isolates in a separate assay. Motility was chosen as a phenotype of interest because it plays an important role in colonization (83) and is thought to play a role in biofilm formation; moreover, biofilm and motility phenotypes have been reported to be linked in some cases, but not others (10, 84–90). Specifically in *H. pylori*, recent studies found that chemotaxis components and flagellar rotation can affect the initial stages of *in vitro* biofilm formation, while deletion of the flagellar gene *fliK* leads to a reduction in biofilm formation and extracellular matrix components (35, 36). In contrast, deletion of flagellar motor cage components such as *pilO* results in increased motility but a defect in microcolony formation and a decrease in biofilm formation (91). Mutation of *pflA* in *H. pylori* G27 leads to non-motile cells that are still flagellated, while mutation of *flgS* results in non-motile and non-flagellated cells (23, 67). Despite the non-motile nature of both of these mutant strains, they displayed opposite biofilm phenotypes in our assay (Fig. S1).

**Fig 4:**
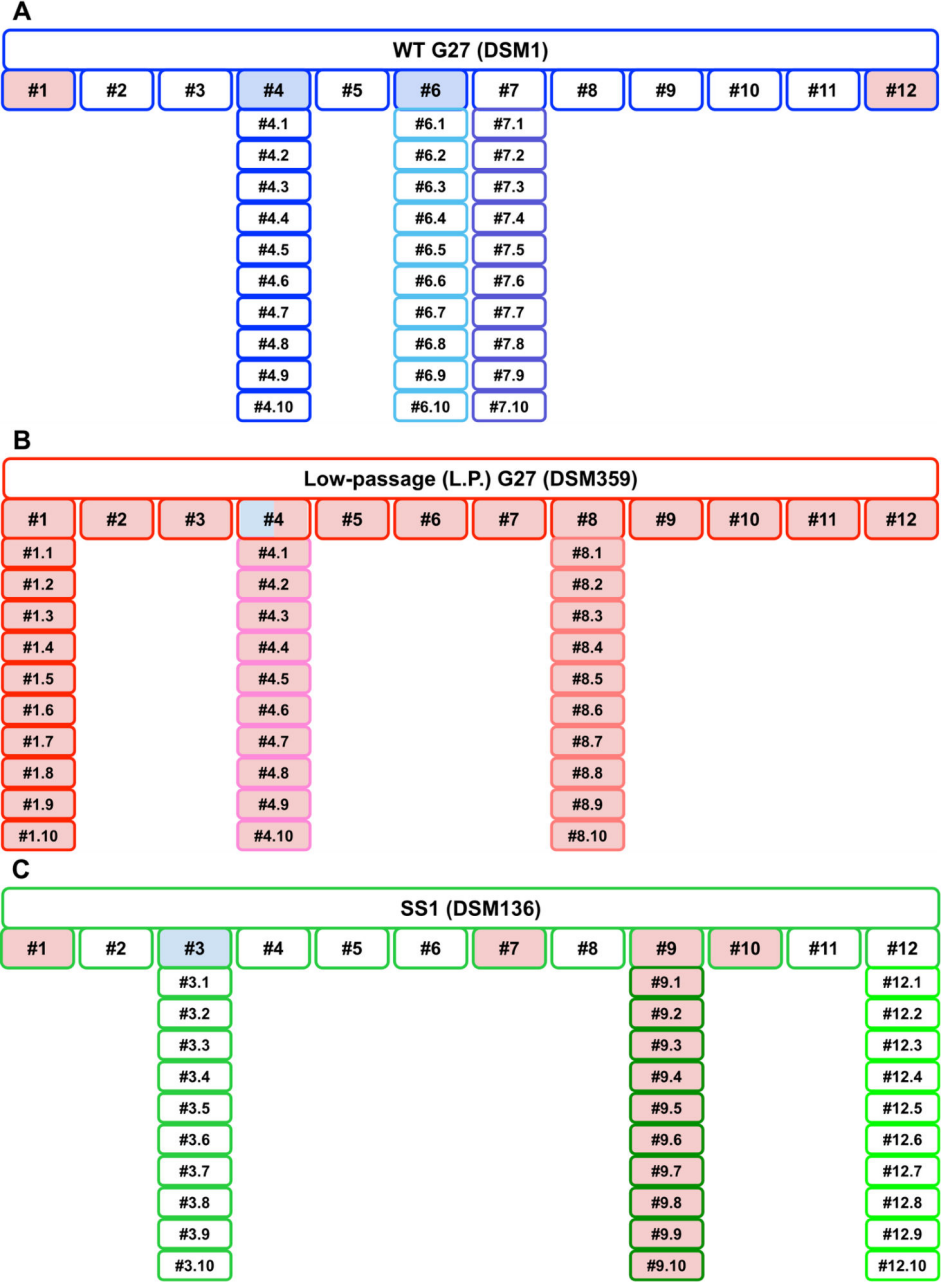
Biofilm and motility phenotypes of all 126 single colony isolates organized by relationship and WT parent strain background. Blue-filled shapes represent isolates with significant biofilm phenotypes as compared to the respective parent strain. Red-filled shapes represent isolates that were non-motile in a soft agar motility assay at day 7 post-inoculation. Shapes that are filled with both blue and red represent isolates with significant biofilm phenotypes that were also non-motile. (**A**) First- and second-generation single colony isolates derived from the WT G27 parent strain (DSM1). (**B**) First- and second-generation single colony isolates derived from the L.P. G27 parent strain (DSM359). (**C**) First- and second-generation single colony isolates derived from the SS1 parent strain (DSM136). Numbers 1–12 indicate the first-generation isolates for each indicated strain background. Second-generation isolates are distinguished by a number designation following the period (e.g., #1.1 indicates second-generation isolate #1 that was derived from first-generation isolate #1 of the indicated parent strain).

To determine whether biomass and motility phenotypes correlated among the single colony isolates from the three *H*. *pylori* parent strains, all 126 isolates (first and second generations) were screened in a soft agar motility assay. Strains were classified as non-motile if a motility halo was not visible by 7 days post-inoculation. Resulting data were then color coded and plotted to simultaneously visualize biofilm and motility phenotypes and their relationships (Fig. 4). Among WT G27 first-generation isolates, motility loss was as frequent as decreased biofilm formation; however, these phenotypes occurred in distinct isolates (#1 and #12 versus #4 and #6, respectively; Fig. 2 and 4A). Loss of motility was even more frequent among SS1-derived isolates; 4 out of the 12 first-generation isolates were non-motile in the soft agar assay (Fig. 4C). Furthermore, none of the second-generation SS1 single colony isolates #9.1–9.10 regained motility.

Similar to the biomass phenotypes (Fig. 3B), L.P. G27 isolates followed different motility trends as well (Fig. 4B). Although the parent stock was motile by day 7 in the soft agar assay, it was slightly delayed in comparison to WT G27 and SS1 (data not shown). However, all tested first- and second-generation L.P. G27 isolates were non-motile. Despite this non-motile status, most of the L.P. G27-derived isolates generally produced more biomass than WT G27 and SS1 isolates (data not shown). Taken together, these data indicate that biofilm and motility phenotypes, as assessed by these two *in vitro* assays, do not correlate in these 126 single colony isolates, regardless of passage history or genetic background of the parent strain.

### Single colony isolates accumulate mutations that could affect biofilm formation

Based on the observed substantial phenotypic variability displayed by the single colony isolates, regardless of strain background, as well as the genetic heterogeneity displayed by the Δ*pflA::cat* isogenic mutant strains, a genomic approach was next used to attempt to identify specific genetic changes that might be responsible for the aforementioned biofilm phenotypes. To this end, based on their patterns of biofilm formation, the following single colony isolates and respective parent strains were chosen for WGS and variant calling analyses: lab WT G27 and G27 #4, #6, and #7; L.P. G27 and L.P. #4, #4.2, #8, and #8.4; SS1 and SS1 #3 and #3.1. Even though the WT G27 lab strain had already been sequenced during the analysis of the G27 Δ*pflA::cat* isolates, the strain was re-sequenced to identify any nucleotide differences between different freezer stocks of DSM1; these two freezer stocks were from the same passage of the strain and were individually frozen from a large-batch liquid culture used to make WT G27 (DSM1) working stocks for the lab. As described above for the Δ*pflA::cat* mutant strains, all sequenced strains and isolates were mapped to the appropriate GenBank reference sequence for the initial variant calling analysis (CP001173.1 or CP009259.1 for G27- or SS1-derived isolates, respectively; Table S2) (69). Next, nucleotide changes were compared between isolates and the matching parent strains to identify changes that were unique to first- and/or second-generation isolates and to attempt to correlate identified changes with relative biomass levels.

**Fig 5:**
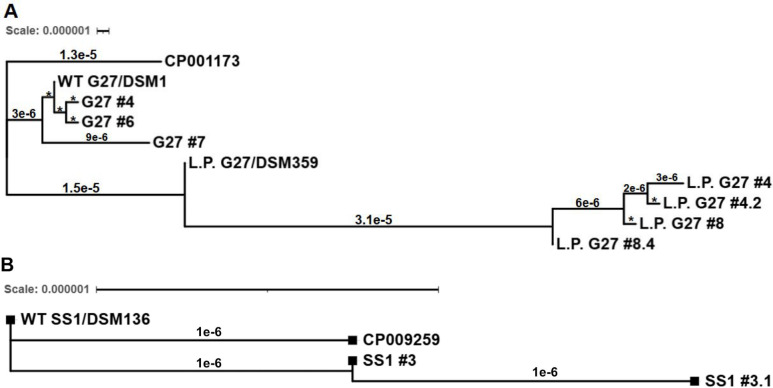
SNP trees were generated to visualize the genetic relationships between sequenced single colony isolates, respective parent strains, and published reference sequences. (**A**) WT G27 (DSM1) and L.P. G27 (DSM359) isolates were compared to the published G27 sequence (CP001173.1) for SNP analysis. (**B**) SS1 (DSM136) isolates were compared to the published SS1 sequence (CP009259.1) for SNP analysis. Branch lengths and the scale bar represent substitutions/site in the genome. Note that although the scale represents 1e-6 (0.000001) in both panels, the scale bar is substantially longer in panel B since there were fewer nucleotide changes between the three sequenced SS1 isolates. Geneious Prime was used to pair, trim, and map Illumina reads from each isolate to the appropriate reference sequence, following the Geneious protocol (92). The Newick file was downloaded for each tree; iTOL (V7) was then used for the final visualization. Some branches in panel A (denoted with *) were too small to be labeled; the length of those branches is 1e-6.

Comparison of the genome sequences for the two independently isolated lab WT G27 (DSM1) gDNA samples revealed only four nucleotide differences, three of which were silent SNPs in *babB* (Tables S1 and S2). Thus, independently patched and expanded populations (i.e., biological replicates) of DSM1 from the same passage were found to be genetically very similar. Furthermore, despite the fact that the lab WT G27 strain had been highly passaged for decades, the WT G27 (DSM1) and L.P. G27 (DSM359) gDNA samples that were sequenced at the same time had similar numbers of nucleotide changes in comparison to the published G27 reference genome (51 and 54 nucleotide differences, respectively; Table S2). Of these changes, only seven nucleotide differences were not shared among these two strains: two were found only in WT G27 (DSM1) and five only in L.P G27 (DSM359). Analysis of the lab-passaged SS1 strain (DSM136) revealed only 17 nucleotide differences as compared to the published SS1 sequence, CP009259.1 (Table S2). However, within just a single lab passage, which takes approximately 10 days (see methods and Fig. S7), the first-generation SS1 isolate that was sequenced (SS1 #3) accumulated seven additional nucleotide changes. Moreover, the second-generation isolate (SS1 #3.1) maintained five of those changes and gained one additional SNP. This general pattern of genetic changes was observed in the other sequenced isolates as well; fewer nucleotide differences were detected in each subsequent generation of isolates. We note that this is also similar to the observation that many second-generation isolates maintained the biofilm or motility phenotypes of their respective first-generation parent strain (Fig. 3 and 4). To visualize patterns of genetic change and divergence, relationship trees that were grouped by the G27 or SS1 lineage were constructed based solely on the number of nucleotide differences identified between the isolates (Fig. 5A and B, respectively). These SNP-based trees generally depicted the ancestral relationships between the isolates; first-generation isolates were more closely related to the parent strain, and second-generation isolates were more closely related to the first-generation isolate. However, as with the biofilm and motility phenotypes (Fig. 3B and 4B), the sequenced L.P. G27 isolates followed a slightly different trend; the second-generation isolates #4.2 and #8.4 were more genetically similar to L.P. G27 than to their respective parent strains, #4 and #8 (Fig. 5A).

The SNP analyses also revealed that the number of differences per site in the genome (shown as branch lengths) was on the order of 10^−6^ to 10^−5^, regardless of the strain background. Although these differences were measured over a single lab passage, the magnitude of change was similar to the estimated mutation rate of *H. pylori* during chronic colonization, ~1.38 × 10^−5^ mutations per site per year (93). Additionally, the rates of genetic change among the single colony isolates in this study were ~10^2^–10^3^-fold higher than mutation rates per generation that have been estimated based on spontaneous resistance to antibiotics *in vitro* (94). This higher rate may be partially explained by the several days of expansion that took place before and after plating the parent strain for single colonies to create freezer stocks of first- and second-generation isolates; a minimum of 10 days of growth took place during this single-passage process (see methods and Fig. S7). Notably, these passage methods differ substantially from those used for a “single passage” of other, faster-growing species; this temporal difference somewhat correlates to the number of accumulated nucleotide changes. As demonstrated by Sabol et al., genera such as *Salmonella*, *Escherichia*, and *Listeria* may only accumulate 3–5 SNPs over the course of 20 *in vitro* passages (61); this is in stark contrast to the dozens of nucleotide changes that were detected over a “single passage” for WT *H. pylori* herein. Taken together, these findings suggest that laboratory growth conditions, expansion, and passage of WT *H. pylori* strains lead to the relatively fast accumulation of nucleotide changes and high levels of genetic heterogeneity within a population of cells.

### Mutation of *sabA*, but not *babA*, *babB*, or *futB*, or the addition of a WT copy of *arsS*, results in a significant decrease in biofilm formation

To determine whether any of the individual nucleotide differences that were identified in the sequenced single colony isolates were responsible for the observed biofilm phenotypes, the list of all affected genes was analyzed to identify rational targets to be studied using a genetic approach; a total of five targets were chosen for further study (Table 1). First, the acid-responsive two-component system encoded by *arsR* and *arsS* was chosen for further study based on the established role of this system in biofilm formation. Specifically, deletion of *arsS* in *H. pylori* G27 results in a hyper-biofilm phenotype (33, 34). Therefore, it was hypothesized that the polynucleotide tract change detected in *arsS* in G27 #7 might decrease *arsS* expression and, therefore, contribute to enhanced biomass production by this first-generation isolate (Table 1). To test this possibility, an *arsS* expression plasmid (pTM117::*arsS*) that was previously shown to complement an *arsS* mutation (33) was transformed into both G27 #7 and the WT parent strain, DSM1; we reasoned that if decreased *arsS* expression was responsible for the increased biomass demonstrated by G27 #7, then increased expression of *arsS* should decrease biofilm formation in this strain. However, this was not the case; the addition of a WT copy of *arsS* instead led to a significant increase in biomass production in both isolates (WT G27 and G27 #7; (Fig. S8). While the reason for this increase remains unclear, these data suggest that decreased *arsS* expression in G27 #7 is not responsible for the augmented biofilm formation demonstrated by this isolate.

**TABLE 1:** Nucleotide changes selected for further study from all WGS and variant calling analyses^*f*^

Isolate with change	Change(s)^*a*^	Predicted outcome^*c*^	Location details^*d*^	Gene^*e*^	Gene product
G27 #7	(C)_15→14_	2 amino acid changes and then an early stop codon	Coding (1,261/1,347 nt)	*HPG27_150*	Histidine kinase sensor protein (ArsS)
G27 Δ*pflA*::*cat* #3	16 SNPs^*b*^	2 amino acid changes, 12 silent substitutions, and 2 double-nucleotide substitutions	Coding	*HPG27_298*	Outer membrane protein (BabA)
G27 #6	(CT)_10→9_	6 amino acid changes and then an early stop codon	Coding (1,920/1,956 nt)	*HPG27_680*	Outer membrane protein (SabA)
G27 #6	(C)_10→11_	10 amino acid changes and then an early stop codon	Coding (67/1,338 nt)	*HPG27_1018*	Alpha-1,3-fucosyl transferase (FutB)

^*a*^ The particular nucleotide change(s) found within the isolate indicated in the previous column; polynucleotide tract length changes are indicated by the nucleotide(s) that are repeated in parentheses followed by the change in repeat number, separated by an arrow; SNP refers to an individual nucleotide substitution.

^*b*^ Additional details about these nucleotide changes can be found in Table S1 under G27 Δ*pflA::cat* #3.

^*c*^ The predicted outcome or consequence of the nucleotide change(s) indicated in the previous column.

^*d*^ The coding sequence position(s) of the nucleotide change(s) is listed out of the total nucleotides (nt) found within that coding sequence.

^*e*^ The specific annotation of the gene found to contain the nucleotide change(s) listed in that row; arrows indicate coding sequence direction.

^*f*^ The nucleotide changes in this table have been edited to reflect the orientation of the genes that contain these changes; Tables S1 and S2 contain the original variant calling outputs and genomic positions of the changes.

**Fig 6:**
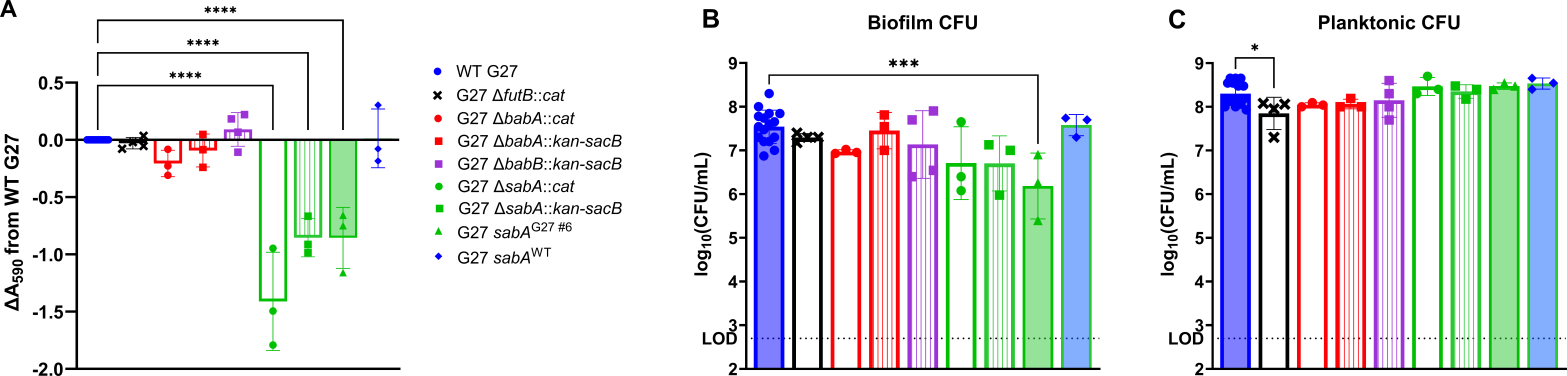
While deletion of *futB*, *babA*, or *babB* in *H. pylori* G27 does not significantly affect biofilm formation at 72 h, deletion or replacement of *sabA* (using multiple antibiotic markers) or insertion of the mutated *sabA* allele from G27 #6 results in a significant decrease in biofilm formation that is alleviated when the G27 #6 *sabA* allele is restored to the WT sequence (*sabA*^WT^). (**A**) OD-controlled liquid overnight cultures were adjusted to an OD_600_ of 0.1 and added to a 24-well plate. After 72 h, the biofilms were washed with brucella broth (BB), allowed to dry, and then stained with 1% crystal violet. Stained biomass was resuspended in an alcohol solution, and the absorbance was read at 590 nm. (**B and C**) A duplicate set of wells was used for planktonic and biofilm CFU enumeration; the limit of detection (LOD, dotted line) was 500 CFU. One-way ANOVAs with Dunnett correction were performed on baseline-corrected (**A**) or log-transformed CFU (**B and C**) data in comparison to WT G27; *n* ≥ 3; individual data points are plotted with the mean and SD; ****P < 0.0001, ***P = 0.0007, and *P = 0.0219 for the indicated comparisons.

Next, the gene encoding an alpha-1,3-fucosyltransferase, *futB*, was also selected for further study based on the presence of a polynucleotide tract change that was identified in the first-generation single colony isolate G27 #6 (Table 1). It was hypothesized that this genetic change could contribute to the loss of biofilm formation by this isolate for several reasons: (i) FutB and other fucosyltransferases have well-established roles in lipopolysaccharide (LPS) biosynthesis and phase variation in *H. pylori*, (ii) the length of this polynucleotide tract in *futB* is known to affect the on/off status of this gene, and (iii) the presence of *futB* is positively associated with biofilm formation in clinical isolates (10, 95–97). Therefore, the predicted off status of *futB* in G27 #6 could have impacted the LPS structure of this isolate and potentially negatively affected biomass production. However, deletion of *futB* in WT G27 (DSM1) did not result in a significant change in biomass production or biofilm CFU (Fig. 6). These data suggest that the *futB* polynucleotide tract change detected in G27 #6 did not contribute to the biofilm defect of this isolate.

The final genes of interest that were selected for further study were *babA*, *babB*, and *sabA*. These genes encode conserved *H. pylori* outer membrane proteins that have been thoroughly defined as adhesins and/or colonization factors but have unknown roles in biofilm formation (7, 51, 72, 98–100). Furthermore, sequence repeats and homologous regions within and upstream of *babA* and *sabA* have been shown to facilitate genetic variation, and these changes have been documented during *in vitro* passage (5). We also noted that multiple nucleotide changes were found in these genes across several of the isolates subjected to WGS and variant calling (Tables S1 and S2). Specifically, *babA* in G27 Δ*pflA::cat* mutant #3 contained over a dozen SNPs with various genetic consequences, including multiple amino acid changes; the occurrence of these secondary changes in the Δ*pflA::cat* strain may have contributed to the loss of the biofilm phenotype upon lab passage of that strain (Fig. S1). In contrast, many of the SNPs identified in *babB* were shared between DSM1, DSM359, and multiple single colony isolates; therefore, deletion of *babB* in G27 was not expected to affect biofilm formation in this assay and served as a control. Last, single colony isolate G27 #6 was found to have a polynucleotide tract change in *sabA* (Table 1). Changes in the length of this polynucleotide tract have been reported to negatively affect *sabA* expression(5, 7, 99, 101, 102); it was hypothesized that a change in *sabA* expression and a corresponding decrease in expression of the encoded adhesin, SabA, might contribute to the biofilm-deficient phenotype of G27 #6. Therefore, deletion of *sabA* (G27 Δ*sabA::cat* or G27 Δ*sabA::kan-sacB*) or insertion of the *sabA*^G27 #6^ allele was expected to have the same effect on biofilm formation in this assay.

To assess the potential effects of *babA*, *babB*, and *sabA* on biomass production, individual deletion mutant strains were constructed, in which the gene of interest was replaced by an antibiotic resistance or antibiotic resistance/counterselection marker. Each of these mutant strains was then tested in the 72 h 24-well crystal violet biofilm assay; biomass was plotted relative to the WT G27 parent strain, DSM1 (Fig. 6). Deletion of *babA* or *babB* in *H. pylori* G27 did not significantly affect biofilm formation, regardless of which antibiotic resistance or antibiotic resistance/counterselection marker was used to replace the gene CDSding sequence (*cat* or *kan-sacB*). However, deletion of *sabA* led to a significant loss in biomass production at 72 h; this phenotype was evident in independently derived strains that were each marked with a different antibiotic resistance cassette (Fig. 6).

To determine if the specific *sabA* allele contained in G27 #6 recapitulated the phenotype of the *sabA* deletion insertion strains, a *sabA* allele-swap strain (G27 *sabA*^G27 #6^) was also constructed by replacing the *kan-sacB* cassette of Δ*sabA::kan-sacB* with the *sabA* ^G27 #6^ allele. This allele-swap strain was significantly biofilm deficient, both by biomass and biofilm CFU measurements (Fig. 6). Furthermore, during the construction of G27 *sabA*^G27 #6^, analysis and sequencing of the locus of interest in multiple isolates revealed a transformant in which the *sabA* allele had been spontaneously restored to the WT sequence (i.e., this isolate no longer contained the polynucleotide tract change identified in G27 #6); this *sabA* restorant strain (G27 *sabA*^WT^) displayed WT levels of biomass and biofilm CFU (Fig. 6). Taken together, these data indicate that while disruption of *babA* or *babB* did not alter biofilm formation of G27, disruption of *sabA* using various approaches resulted in a significant decrease in biofilm formation. Furthermore, the identified *sabA* polynucleotide tract change found within G27 #6 was sufficient to decrease biofilm formation in this strain. This later finding strongly indicates that the polynucleotide tract change identified in G27 #6 was responsible for the decreased biofilm formation exhibited by this isolate.

## DISCUSSION

By critically assessing the phenotypic and genotypic changes that occur during the construction of “isogenic” mutant strains and laboratory passage of *H. pylori*, here, we demonstrate that the high levels of genetic heterogeneity and plasticity in *H. pylori* populations can complicate the interpretation of subsequent phenotypic results. This phenomenon was found within a constructed mutant strain (G27 Δ*pflA::cat*) as well as in WT laboratory-passaged strains: high-passage and L.P. G27 isolates and the commonly studied *H. pylori* mouse-colonizing strain, SS1 (Fig. 2–4). Assessment of individual WT single colony isolates revealed that the level of phenotypic variability was affected by the phenotype being assessed. For example, ~11% of WT first-generation single colony isolates showed significant changes in biofilm phenotypes, but 50% of these same isolates displayed motility phenotypes (Fig. 2 and 4). How this variability would affect other phenotypes of interest would require direct assessment; however, this finding highlights the critical necessity of creating and/or analyzing multiple biologically independent isolates/transformants when assessing any phenotype of interest in *H. pylori*. The same is true even when using complementation, particularly when a loss of function has been identified. Additionally, WGS of newly constructed and/or screened strains may be necessary to rule out changes that could be responsible for the phenotype of interest. Indeed, many nucleotide changes were identified in the sequenced G27 Δ*pflA::cat* mutant isolates that may have contributed to the variability in biomass displayed by these strains that were expected to be “isogenic” (Table 1). Of note, only one nucleotide change was shared among all three mutant isolates that were sequenced (Δ*pflA::cat* #1, #3, and #6); all other changes were unique to only one or two of the three isolates (Table 1). While this may indicate randomness in the accumulation of these genetic changes, the loss of the hyper-biofilm phenotype of the original G27 Δ*pflA::cat* mutant strain may indicate that there is an *in vitro* fitness cost associated with overproduction of the extracellular polymeric substance (EPS) components of the biofilm that may impact the quantity and diversity of these nucleotide changes. This potential *in vitro* fitness cost is also supported by the relatively frequent loss of WT or elevated biomass production that was observed in the analysis of the WT first- and second-generation isolates (Fig. 2 and 3); decreased biomass was more common than increased biomass, and strains that displayed increases tended to lose this phenotype upon subsequent passage (Fig. 3).

From the list of specific nucleotide changes that were identified in isolates showing a change in biomass, the adhesin-encoding gene *sabA* was identified as a locus that was potentially involved in biofilm formation; nucleotide changes identified in G27 #6 correlated with a significant decrease in biomass production in this isolate (Fig. 2; Table S2; Table 1). Subsequent downstream genetic analysis of multiple independently constructed strains confirmed that the polynucleotide tract change in *sabA*^G27 #6^ was sufficient to decrease biofilm formation of G27 (Fig. 6). These results are in keeping with prior studies that showed that a change in this polynucleotide tract is associated with decreased SabA expression (99, 102); although protein expression changes were not measured herein, it is likely that the same phenomenon is at play. The biofilm deficiencies of both *sabA* knockout strains further support the idea that expression of this protein is important for *in vitro* biofilm formation by strain G27 in our assay. Although the deletion of other targets (i.e., *babA*, *babB*, and *futB*) did not result in a significant change in biofilm formation, this study is the first to report any biofilm phenotypes, or lack thereof, for these four genes in *H. pylori*, which are otherwise relatively well studied.

Beyond biofilm phenotypes, while further investigating specific nucleotide changes identified in G27 #6, we attempted to transform this single colony isolate with complementation vectors and repeatedly recovered few to no colonies (data not shown). To quantify this potential defect, we tested the transformation efficiency of G27 #6, a related second-generation isolate with a similar biomass level, G27 #6.9, and another first-generation isolate that was also biofilm deficient, G27 #4, as compared to WT G27; gDNA, a linear PCR product, and plasmid DNA (pTM117) were each used for transformation (Fig. S9). Both G27 #6 and #6.9 showed significantly decreased transformation efficiencies for both gDNA and a linear PCR product; this trend was similar, albeit not significant, for plasmid DNA as well. Despite this transformation deficiency, variant calling analysis of G27 #6 did not reveal any nucleotide changes in genes that are known to be involved in transformation and/or natural competence of *H. pylori* (Table S2) (103–105). Although not all of the first- and second-generation single colony isolates were screened for transformation efficiency defects (as was done for biofilm and motility phenotypes), these data further demonstrate that the genetic heterogeneity within WT *H. pylori* populations might affect numerous phenotypes, including natural competence, but the specific nucleotide changes that contribute to these phenotypes can be difficult to identify.

In thinking about the WGS and variant calling analyses conducted on the Illumina platform and using *breseq*, it is important to note that a cutoff of 80% was used to call mutations. In other words, a SNP was called as a variant if 80% or more of the reads at that nucleotide did not match the reference sequence. This means that at the time of gDNA isolation, up to 20% of the reads could have contained a different sequence that may or may not match the reference sequence. It is unknown whether this percentage translates directly to genetic variability in the population or if 20% is a biologically relevant percentage of that population for *H. pylori*. However, this point helps to emphasize that in the age of next-generation sequencing, more research is needed to determine the biological significance of these cutoffs. Furthermore, we note that biological relevance could greatly depend on the bacterial species being studied and the differing background levels of genetic heterogeneity found within these species.

In order to partially address the open question of biologically relevant cutoffs for variant calling, we conducted a marginal call analysis where nucleotide variants that were present at a frequency between 30% and 80% in each parent strain (DSM1, DSM359, or DSM136) were tracked in the respective first- and second-generation single colony isolates that were sequenced (Table S3). This allowed for the identification of nucleotide changes that were present at a frequency <80% within the respective parent gDNA sample. Many changes were identified, some of which were unique to a single isolate, while others were shared among all sequenced isolates derived from a given parent strain (Table S3). Furthermore, dozens of other variants appeared with frequencies <30%; this cutoff was chosen rather than a lower percentage because it provided a reasonable number of mutations to track across the sequenced first- and second-generation isolates. However, it is worth noting that it is also unknown if this is a biologically appropriate cutoff. Despite this caveat, when critically assessing the strain passage and construction methods used for *H. pylori*, these variant frequencies are relevant as a measure of overall population genetic heterogeneity. This is particularly true where single colonies are selected, expanded, and then screened for a genotype and phenotype of interest (Fig. S7). In this scenario, nucleotide variant frequency across reads could coincide with the likelihood of selecting and expanding a single colony isolate with that particular mutation. Thus, the high number of marginal calls identified during WGS could partially explain the biofilm and motility variation in multiple mutant and WT *H. pylori* strain backgrounds assessed in this study.

Of note, it is important to acknowledge that our conducted downstream SNP analyses focused on the *H. pylori* chromosome and annotated CDSs; we did not include variant calling of the *H. pylori* plasmid or a detailed promoter analysis, which may have identified additional genetic changes associated with particular biomass phenotypes. Furthermore, there is a growing body of research that supports the role of small RNAs in *H. pylori* virulence factor regulation (106–110), but investigation of these genetic components was considered outside the scope of this study. Thus, a deeper dive into the data sets generated here may reveal additional sequences of interest.

In addition to the biomass differences and genetic variability of WT single colony isolates, we found that loss of motility was relatively frequent among first-generation single colony isolates derived from DSM1 (WT G27) and DSM136 (SS1); twice as many SS1 first-generation isolates lost motility in the soft agar assay as compared to G27 (Fig. 4). Notably, a loss of biofilm formation occurred at a similarly high frequency among single colony isolates (Fig. 3); though the frequent loss of motility has been noted (111), the relatively frequent loss of biofilm formation *in vitro* has not been previously reported for *H. pylori*. Furthermore, despite being conditionally associated in other species, motility and biofilm phenotypes did not correlate in these *H. pylori* single colony isolates, regardless of strain background; strains that were non-motile were not necessarily biofilm deficient and vice versa. Since motility was assessed secondary to biofilm formation, none of the non-motile DSM1- or DSM136-derived single colony isolates were chosen for sequencing and variant calling analysis; thus, the reason(s) for loss of motility in these isolates is currently unknown. It is also unclear why none of the first- or second-generation L.P. G27 isolates were motile by day 7 in the soft agar motility assay; the parent strain, DSM359, was motile by that time point. Although this finding suggests that the majority of the DSM359 population had delayed motility or was non-motile, SNP analyses did not reveal any shared nucleotide changes among L.P. G27 #4, #4.2, #8, and #8.4 that might have led to the further decreased motility of these isolates (Table S2). Notably, the binary “yes/no” observations recorded during this screen did not allow for the assessment of subtle changes in motility over time (see methods); more detailed motility assays would be needed to further investigate this complex phenotype. However, the frequent loss of motility among first-generation single colony isolates is even more confounding when considering that (i) motility was not restored in any of the L.P. G27 isolates or in the SS1 #9.1–10 second-generation isolates and (ii) loss of motility did not occur in any of the WT G27 (DSM1) or SS1 (DSM136) second-generation isolates. These observations, respectively, suggest that (i) irreversible genetic change(s) most likely led to the loss of motility seen in first-generation isolates and (ii) second-generation isolates were more likely to maintain the motility phenotype of the respective first-generation parent strain. These findings also apply to the biofilm phenotypes of these isolates.

It is possible that the long-term, population-level passage of laboratory strains, such as DSM1, leads to a relatively stable but diverse genetic pool that may still undergo a mutational “burst” upon the *in vitro* selection and expansion of single colonies. Although not yet described for *H. pylori*, it is well known that diversity within bacterial populations and environments like biofilms facilitate cellular cooperation and the survival of “cheaters” within the population (112–114); therefore, it is possible that an *H. pylori* “cheater” cell that was being supported by the other members of the population may need to accumulate additional genetic changes upon single colony selection in order to survive/thrive. Ultimately, the genetic variation detected during passaging of WT *H. pylori* isolates in this study significantly affected two uncorrelated, multigenic phenotypes that are central to *H. pylori* pathogenesis research. The multigenic nature of these phenotypes undoubtedly increases the likelihood of a genetic change affecting that phenotype. Additional studies may reveal a difference in the frequency of phenotypic variability when less complex processes are assessed, specifically those that are monogenic. Failure to study any monogenic phenotypes is a limitation of the current study. Similarly, we acknowledge that after we observed the inconsistent biofilm phenotypes of the six new *pflA* mutant strains (Fig. 1), we focused our efforts on characterizing/understanding the role of *H. pylori* heterogeneity on downstream analyses and did not complement the *pflA* and *flgS* mutations that we originally set out to study (Fig. 1; Fig. S1). Based on the observations herein, redundant mutant and complementation strain construction will be needed to definitively connect these genotypes to the observed phenotypes.

**Fig 7:**
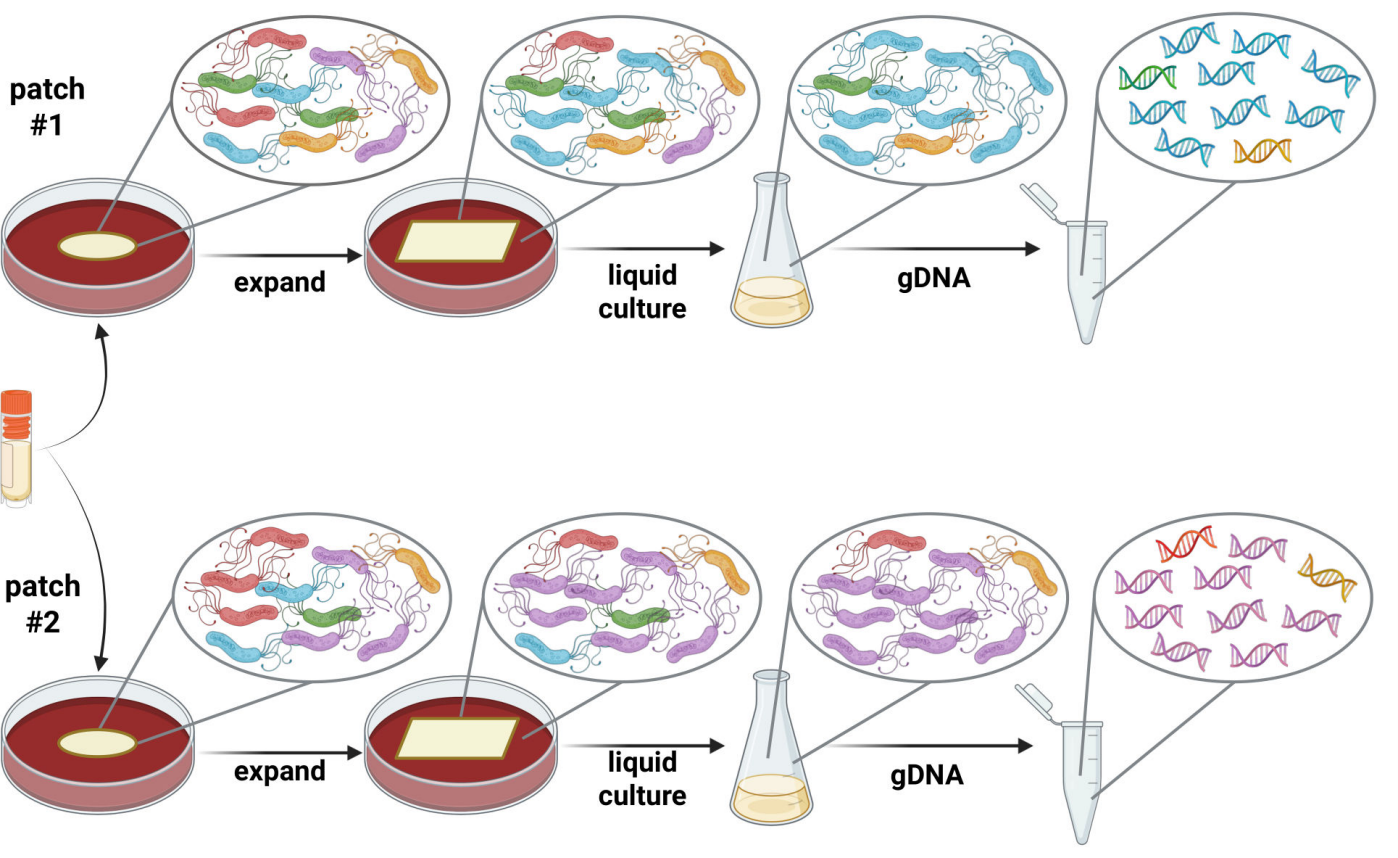
Model of genetic heterogeneity within *H. pylori* cultures during growth and expansion. During normal laboratory expansion, *H. pylori* cultures are grown for several days on both solid agar plates and in liquid media, which allows the bacteria to accumulate nucleotide changes, undergo phase variation, and diversify. While this may occur stochastically, many factors and microenvironments might affect this process, despite apparently identical expansion methods/media conditions and the lack of an obvious selection. This can ultimately lead to unique genetics between WT populations that originated from the same freezer stock; heterogeneity can be detected during WGS, read mapping, and variant calling analyses. In this model, patch #1 and #2 both contain individual isolates that have various nucleotide changes within their genomes; these are represented by the individually colored *H. pylori* cells, where each color indicates a different combination of nucleotide changes. During outgrowth, stochastic events, unknown selective pressures, ongoing phase variation, etc. may result in changes in the population structure such that particular isolates become more common at various stages. For example, the scenario for patch #1 indicates that the “blue” isolate becomes a major proportion of the population; if the liquid culture was plated for single colony isolation, colonies with the “blue” combination of alleles would be most likely to be isolated. Similarly, if gDNA was prepared and sequenced, and the standard 80% frequency within the sequencing reads cutoff was used, the obtained “blue” sequence would be identified as the genomic sequence for the strain. Alternatively, for patch #2, the “purple” isolate becomes most abundant and would be more likely to be chosen for expansion; genomic sequencing would reveal the “purple” sequence as the genomic sequence for the strain. Created in BioRender (available from https://BioRender.com/18x5wds).

Taken together, the results described herein indicate that even normal laboratory passage can lead to heterogeneity within and between *H. pylori* populations, despite the use of identical methods and lack of an obvious selective pressure; a model for how this could occur is presented in Fig. 7. *H. pylori* grows slowly, and cultures are typically grown for several days, which allows the bacteria to accumulate nucleotide changes, undergo phase variation, and diversify. While this may occur stochastically, many different factors and minute environmental changes might also affect this process, despite seemingly identical expansion methods and media conditions. As such, these changes can ultimately lead to unique genetics between WT populations that originated from the same freezer stock (Tables S1 and S2). Additionally, unknown epistatic interactions between genes and/or the occurrence of compensatory genetic changes might further complicate the background genetics. In the proposed model, patch #1 and patch #2 both contain individual isolates that have various nucleotide changes within their genomes; these are represented by the individually colored *H. pylori* cells, where each color indicates a different combination of nucleotide changes. During outgrowth, changes in the population structure may occur such that particular isolates become more common at various stages. For example, if gDNA was prepared and sequenced, and the standard 80% frequency within the sequencing-reads cutoff was used, the obtained “blue” sequence would be identified as the genomic sequence for the strain from patch #1. Alternatively, for patch #2, the “purple” isolate becomes most abundant and would be more likely to be chosen for expansion; likewise, sequencing would reveal the “purple” sequence as the genomic sequence for the strain.

Beyond effects on any given lab’s individual results, these findings also indicate that WT strains that are found within different labs may be genetically different; laboratory passage protocols of individual research groups may lead to different nucleotide changes in WT *H. pylori* stocks, producing strains that are genetically distinct from those used in this study (e.g., DSM1). One could imagine how this could affect the reproducibility of experimental outcomes across laboratories, which has been recognized as a significant issue across scientific research (115–117). Overall, it is critical to consider the inherent phenotypic variability of *H. pylori* (and perhaps other bacterial species), particularly when investigating loss of function and complex phenotypes that might be affected by numerous genes and mutations. In fact, independently targeting multiple related factors within a single biological pathway could provide additional evidence for a particular phenotype. Last, although this study demonstrated that WGS and downstream analyses can be used to compare *H. pylori* isolates, identify mutations of interest, correlate mutation presence to phenotypes of interest, and monitor the high levels of genetic heterogeneity within *H. pylori* populations, additional biologically independent studies are absolutely necessary to determine the relevance of the outputs from these genomic methods to any phenotype. In summary, our results underscore the necessity for redundancy and/or complementation when attempting to attribute the action of a particular gene to a particular phenotype or even ascribe a phenotype to a WT strain of interest.

## MATERIALS AND METHODS

### Bacterial strains and growth conditions

Table S4 lists the strains, primers, and plasmids used in this study. Liquid brucella broth (BB) (Neogen Corporation) media supplemented with 10% FBS (Gibco) and 10 µg/mL vancomycin (BB10) or horse blood agar (HBA) plates containing 4% Columbia agar (Neogen Corporation), 5% defibrinated horse blood (HemoStat Laboratories, Dixson, CA), 2 mg/mL β-cyclodextrin (Sigma-Aldrich), and an antibiotic/antifungal cocktail composed of 10 µg/mL vancomycin (Amresco), 5 µg/mL cefsulodin (Sigma-Aldrich), 2.5 U/mL polymyxin B (Sigma-Aldrich), 5 µg/mL trimethoprim (Sigma-Aldrich), and 8 µg/mL amphotericin B (Amresco) were used to culture all *H. pylori* strains. Where needed for mutant strain construction, growth media were supplemented with 20 or 25 µg/mL kanamycin (Kan20 or Kan25; Gibco), 10% sucrose (S10%; Sigma-Aldrich), or 6 or 8 µg/mL chloramphenicol (Cm6 or Cm8) (Sigma). *H. pylori* stock cultures were made using freezing media (brain heart infusion broth [BD], 10% FBS, and 20% glycerol [EMD Chemicals, Inc.]) and were stored at −80°C. In general, *H. pylori* strains were cultured as described in Carpenter et al., 2007 (118). Gas evacuation jars were used to culture all *H. pylori* strains at 37°C and under microaerobic conditions (5% O_2_, 10% CO_2_, and 85% N_2_) that were achieved using an Anoxomat system (Advanced Instruments, Inc.). Liquid cultures were grown with shaking at 110 RPM.

For our experiments, one laboratory passage of the *H. pylori* strains was considered to be the length of time needed to expand a strain of interest from the freezer until archiving of individual single colony isolates as new freezer stocks, as depicted in Fig. S7; this was approximately 10 days of total growth in the indicated conditions. For most WT strains, each step depicted in Fig. S7 occurred after 18–24 h. In some cases, including for SS1-derived isolates, strains needed an extra day of growth after being patched from the freezer stock; growth over subsequent steps was then comparable across strains.

*Escherichia coli* Top10 cells were used to maintain plasmids for *H. pylori* mutagenesis. These cells were grown in lysogeny broth (LB; Lennox, Invitrogen) or on LB agar (Miller, Thermo Scientific) plates supplemented with 100 µg/mL ampicillin (Amp100; Sigma-Aldrich) or 25 µg/mL kanamycin (Kan25), as appropriate for each plasmid. *E. coli* cultures were grown with shaking at 190 RPM, and freezer stocks were made by adding 80% glycerol (Acros Organics) to an overnight culture at a 1:1 ratio and stored at −80°C.

### Strain construction

In most cases, *H. pylori* G27 (DSM1) mutant strains were constructed, and transformations were carried out as previously described by Servetas et al. (119). Both the G27 Δ*flgS::cat* and original Δ*pflA::cat* clean deletion insertion mutant strains (DSM1098 and DSM709, respectively) were constructed by fusing the regions upstream and downstream of each CDS (*HPG27_224* and *HPG27_1219*, respectively) to the chloramphenicol resistance cassette (*cat*), cleaning the final splicing by overlap extension (SOE) PCR product (Qiagen QIAquick PCR Purification Kit), transforming that linear product into WT G27 (DSM1), and selecting on HBA + Cm8 plates. The newly constructed G27 Δ*pflA::cat* mutants #1–6 (DSM2246–DSM2251) were isolated from a single experiment, where a cleaned linear PCR product spanning the Δ*pflA::cat* locus (amplified from DSM709 gDNA) was transformed into DSM1, and transformants were selected on HBA + Cm8. The G27 Δ*futB::cat*, Δ*babA::cat*, Δ*babA::kan-sacB*, and Δ*sabA::cat* mutant strains (DSM2381, DSM2382, DSM2383, and DSM2391, respectively) were constructed similarly to G27 Δ*flgS::cat* (DSM1098); SOE PCR was used to fuse the upstream and downstream regions of each CDS (*HPG27_1018*, *HPG27_298*, and *HPG27_680*, respectively), ending just before the start codon and starting immediately after the stop codon, respectively, to the indicated antibiotic resistance cassette; a linear product was then transformed into DSM1, and transformants were selected as above.

Construction of the G27 Δ*babB::kan-sacB* and Δ*sabA::kan-sacB* strains (DSM2390 and DSM2394, respectively) involved a different approach. Briefly, the regions upstream and downstream of *babB* or *sabA* (*HPG27_1187* or *HPG27_680*, respectively), ending just before the start codon and starting immediately after the stop codon, respectively, were fused using SOE PCR primers that added XhoI (CTCGAG) and SmaI (CCCGGG) restriction enzyme cut sites (CS) between the regions. This linear product was cleaned, A-tailed, cloned into pGEM-T easy (Promega), and transformed into *E. coli* Top10 cells (e.g., pGEM T-easy:*babB* up/CS/down). After PCR confirmation of the expected-sized product with pGEM T-easy SP6 and T7 primers, these vectors were isolated from 5 mL overnight LB + Amp100 cultures (Qiagen QIAprep Spin Miniprep Kit), digested with XhoI and SmaI, cleaned (Qiagen MinElute Reaction Cleanup Kit), and ligated with similarly digested and gel-purified *kan-sacB* that had been liberated from pKSF-II via double digestion. This ligation was transformed into *E. coli* Top10 cells for plasmid maintenance (e.g., pGEM T-easy:*babB* up/*kan-sacB*/down). Minipreps of pGEM T-easy:*babB* up/*kan-sacB*/down and pGEM T-easy:*sabA* up/*kan-sacB*/down were used for PCR confirmation and then individually transformed into WT G27 (DSM1); transformants were selected on HBA + Kan20, and resulting gDNA preparations from each were confirmed via PCR with verification primers that flank the upstream/downstream regions used in the constructs. Phusion Hot Start II DNA polymerase was used for all PCR steps (Thermo Scientific). Multiple PCR-confirmed transformants of each mutant strain were screened in the biofilm assay; in all cases, representative data are shown for individual tested isolates.

To construct the *sabA* allele-swap strain (DSM2395, G27 *sabA*^G27 #6^), gDNA from G27 #6 was used as template to amplify the *sabA* CDS and flanking upstream/downstream regions. This linear PCR product was cleaned and then transformed into G27 Δ*sabA::kan-sacB* (DSM2394); transformants were selected on HBA + S10% and then patched onto HBA + Kan20 to confirm kanamycin sensitivity. Several sucrose-resistant, kanamycin-sensitive, and PCR-confirmed transformants were isolated from multiple biologically independent transformations; however, after sequencing the *sabA* alleles of two isolates from one of these transformations, only one strain maintained the desired *sabA* polynucleotide-tract mutation from G27 #6—the biofilm data from this G27 *sabA*^G27 #6^ isolate (DSM2395) and the isolate that reverted to the WT *sabA* allele (DSM2396, G27 *sabA*^WT^) are shown in Fig. 6.

To construct the *arsS*-related strains, pTM117 (empty vector, isolated from DSM199) and pTM117::*arsS* (isolated from DSM1525) were individually transformed into DSM1 and single colony isolate G27 #7 (33, 118). Transformants were selected on HBA + Kan20, and minipreps were used to confirm the transformants using pTM117- and/or insert-specific primers.

### Biofilm assay

Biofilms were grown and analyzed as previously described by Wylie et al. (27). Briefly, overnight liquid cultures were adjusted to an OD_600_ of 0.1 by pelleting the required volume of cells and resuspending in fresh BB10 media or BB containing 1% FBS in the case of WT SS1 (DSM136) and SS1-derived single colony isolates. Next, 1 mL of each suspension was added in duplicate wells to a 24-well tissue culture-treated plate (Corning Costar). Using the Anoxomat system, the plates were incubated in microaerobic conditions at 37°C with shaking at 110 RPM for 72 h. At this 3-day time point, the duplicate wells were divided into two groups for quantification: CFU and biomass. For enumeration, the media was first removed from the CFU-assigned wells to collect planktonic bacteria. Aggregation was prevented/disrupted by supplementing the samples with 2 µM EDTA and sonicating for 10 min in a water bath (Branson 1510). Planktonic CFU samples were then diluted and plated onto HBA plates for incubation and enumeration 3 days later. The same set of wells was next washed twice with BB10 to remove any remaining planktonic cells; 1 mL of BB10 supplemented with 200 µg/mL proteinase K (Sigma-Aldrich) was next added to each well to remove the biofilm-associated cells from the plate surface. Following a 1 h incubation with shaking, the 1 mL biofilm CFU cultures were treated with 2 µM EDTA and sonicated for 10 min to break up any cellular aggregates. Samples were then diluted and plated onto HBA for later enumeration. The limit of detection for CFU quantification was 500 CFU.

The duplicate wells were used to quantify biofilm biomass. For these wells, the media was removed, and the wells were washed twice with PBS before being allowed to air dry. To stain the biomass, 2 mL of 1% Gram’s crystal violet solution (Sigma, diluted with water) was added to each well, and the plate was incubated at room temperature for 15 min. After staining, the wells were washed three times with distilled water and allowed to air dry. Crystal violet-stained biomass was fully solubilized using 1 mL of differentiation solution (Sigma) per well. Finally, the absorbance of each solubilized sample was read at 590 nm (A_590_) as a measure of biomass. The data displayed and analyzed here represent the results from three or more biologically independent experiments.

### Soft agar motility assay

Strains were patched onto HBA plates and expanded onto fresh HBA plates the following day. After incubation overnight, a sterile pipette tip was used to scrape across the lawn and then stab the soft agar motility plate; these BB plates contained 0.35% agar (Thermo Scientific), 2.5% FBS, and the same *H. pylori*-selective antibiotic/antifungal cocktail as described above. The plates were each poured to contain 30 mL of media and were allowed to dry at room temperature for 3 days before inoculation. At least two stabs per strain (technical replicates) per biological replicate were tested, and each strain was assessed in biological duplicate or triplicate. The plates were left undisturbed for 5 days before the first observation; motility halos were checked again on day 7. If a round motility halo was visible after 7 days of incubation for at least one of the technical replicates of each biological replicate, the strain was deemed motile.

### Transformation efficiency

Strains were patched from the freezer stock and grown as described above. The lawn was collected, resuspended in BB10, and concentrated into a small volume (100–200 µL). For each transformation, 50 µL of concentrated cells was spotted onto fresh HBA plates, allowed to dry, and then incubated microaerobically for 3–4 h. At that point, the DNA samples, each of which contained a kanamycin resistance cassette (*kan*), were added to an individual spot of cells, and the transformation was incubated overnight. A total of 500 ng of DNA per transformation was used for plasmid (pTM117 isolated from DSM199) and gDNA (obtained from DSM2462 [G27 Δ*436::kan*]) samples, while 250 ng was used for the linear DNA transformation; this linear DNA represented an approximately 2 kb cleaned (Qiagen QIAquick PCR Purification Kit) PCR product obtained using primers *436*_F_up and *436*_R_down and template DNA from DSM2462. The next day, the spot of cells was swabbed from the plates, resuspended in 1 mL BB10, serially diluted, and spot plated onto both HBA and HBA + Kan25 plates. After 3 or 4 days, colonies were enumerated to determine total CFU (HBA without antibiotic selection) and CFU of transformants (HBA + Kan25). The number of transformants was divided by the total CFU and then multiplied by 100 to obtain percent transformants. This percentage was then divided by the amount (ng) of DNA used in that particular transformation to yield the percentage of transformants/ng of DNA. A minimum of four biological replicates was conducted with each DNA sample type and each strain of interest.

### Statistical analyses

All CFU data were log-transformed before analysis and plotting. GraphPad Prism (version 10.4.1) was used to perform all statistical analyses. For each data set, a one- or two-way ANOVA was done to compare data to a single control sample; except where noted, the control sample was the respective parent strain for each group of strains or isolates. To account for the typical variability in biofilm formation between replicates, particularly for strains that produce higher levels of biomass, most biomass data are plotted as normalized to the respective parent (control) strain that was grown on the same plate; as a reference, Fig. 1 and Fig. S1 display non-normalized biomass data for WT G27 and several mutant strains. As recommended by Prism, Bonferroni or Dunnett correction for multiple comparisons was used for one- and two-way ANOVAs; means are plotted with individual data points, and the error bars represent standard deviation.

### Single colony isolation

A visual depiction of the following methods can be found in Fig. S7. To obtain first-generation single colony isolates from DSM1 (WT G27), DSM359 (L.P. G27), and DSM136 (SS1), each freezer stock was patched onto HBA. The next day, the patch was expanded onto one half of a fresh HBA plate. On day 3, the expanded lawn was collected with a sterile cotton swab and was used to inoculate a 5 mL liquid culture of BB10 in a 25 mL flask. This liquid culture was then grown with shaking for 18–20 h before being diluted 1:1,000 in BB10; 1 µL of the diluted sample was then spread onto duplicate HBA plates to yield two plates per strain. Single colonies became visible after ~3 days of incubation. At this point, at least 12 well-isolated single colonies per strain were picked and individually streaked onto new HBA plates. After 2 days, the single colony isolate patches were expanded onto fresh plates to larger patches/lawns. The next day, the lawned single colony isolates were used to start overnight liquid cultures in 5 mL of BB10. After 18–20 h of growth, 12 concentrated freezer stocks were created per strain, for a total of 36 first-generation single colony isolates.

To obtain second-generation single colony isolates, the steps described above were repeated beginning with the freezer stocks of selected first-generation isolates: G27 #4, #6, and #7, L.P. G27 #1, #4, and #8, and SS1 #3, #9, and #12. These isolates were chosen due to their relative biomass differences from their respective parent strain. These differences were either statistically significant or the isolate was chosen to represent one of three biomass phenotypes: similar to or slightly higher than respective parent strain levels, trends higher than the parent strain, or trends lower than the parent strain.

### Sequencing and variant calling

The rationale for WGS of the selected isolates was based on relative biomass differences in comparison to the respective parent strain. The three parent strains (DSM1, DSM359, and DSM136) were sequenced for two reasons: (i) to have a recent genome sequence to compare the related first- and second-generation isolates and (ii) to compare the WT lab strains to the corresponding previously published sequences (available from NCBI) (63, 70). The first-generation single colony isolates (as described above) were selected for WGS for the same reasons that they were selected for second-generation isolation, and the second-generation single colony isolates that were sequenced were selected for similar reasons.

gDNA samples (Wizard Genomic DNA Purification Kit, Promega) were sent to SeqCenter, where libraries were prepped (Illumina DNA Prep kit) and sequenced (Illumina NextSeq 2000 or NovaSeq 6000). Average sequencing coverage was 479× (range: 144×–1,286×) across strains, and 91%–96% of bases exceeded the Q30 threshold. On average, reads were 147 bases in length, and 90% of reads mapped to the respective reference genome for each strain. *breseq* (version 36.1 or 38.1) was used for downstream variant calling (69). The sequencers and software programs used correspond to the first and second rounds of WGS and variant calling that were performed for the G27 Δ*pflA::cat* mutant strains and the single colony isolates, respectively.

For SNP tree generation, Illumina reads were imported into Geneious Prime (version 2023.0.4), paired, trimmed using BBDuk, and mapped to the appropriate reference sequence (CP001173.1 or CP009259.1 for G27- or SS1-derived isolates, respectively) (92). Newick tree files were downloaded from Geneious; iTOL (v7) was used for the final visualization.

## Data Availability

Reads from the whole genome sequencing conducted as part of this study can be found at BioProject PRJNA1283251.

## SUPPLEMENTAL MATERIAL

Supplemental figures msphere.00638-25-s0001.pdf Figures S1 to S9.

Table S1 msphere.00638-25-s0002.xlsx All nucleotide changes from WT G27 (DSM1) and G27 Δ*pflA*::*cat* #1, #3, and #6 WGS.

Table S2 msphere.00638-25-s0003.xlsx All nucleotide changes from indicated single colony isolate WGS.

Table S3 msphere.00638-25-s0004.xlsx All marginal calls in sequenced single colony isolates and parent strains, as identified by *breseq*.

Table S4 msphere.00638-25-s0005.xlsx Strains, plasmids, and primers used in this study.

ASM does not own the copyrights to Supplemental Material that may be linked to, or accessed through, an article. The authors have granted ASM a non-exclusive, world-wide license to publish the Supplemental Material files. Please contact the corresponding author directly for reuse.
