# Supplementary material for: Correlation versus causation: Helicobacter pylori population heterogeneity complicates the identification of mutant strain phenotypes
Source: mSphere. 2025 Dec 15;11(1):e00638-25. doi: 10.1128/msphere.00638-25 (PMC12838425; doi:10.1128/msphere.00638-25)
Supplement: Supplemental figures — Figures S1 to S9. [file msphere.00638-25-s0001.pdf]

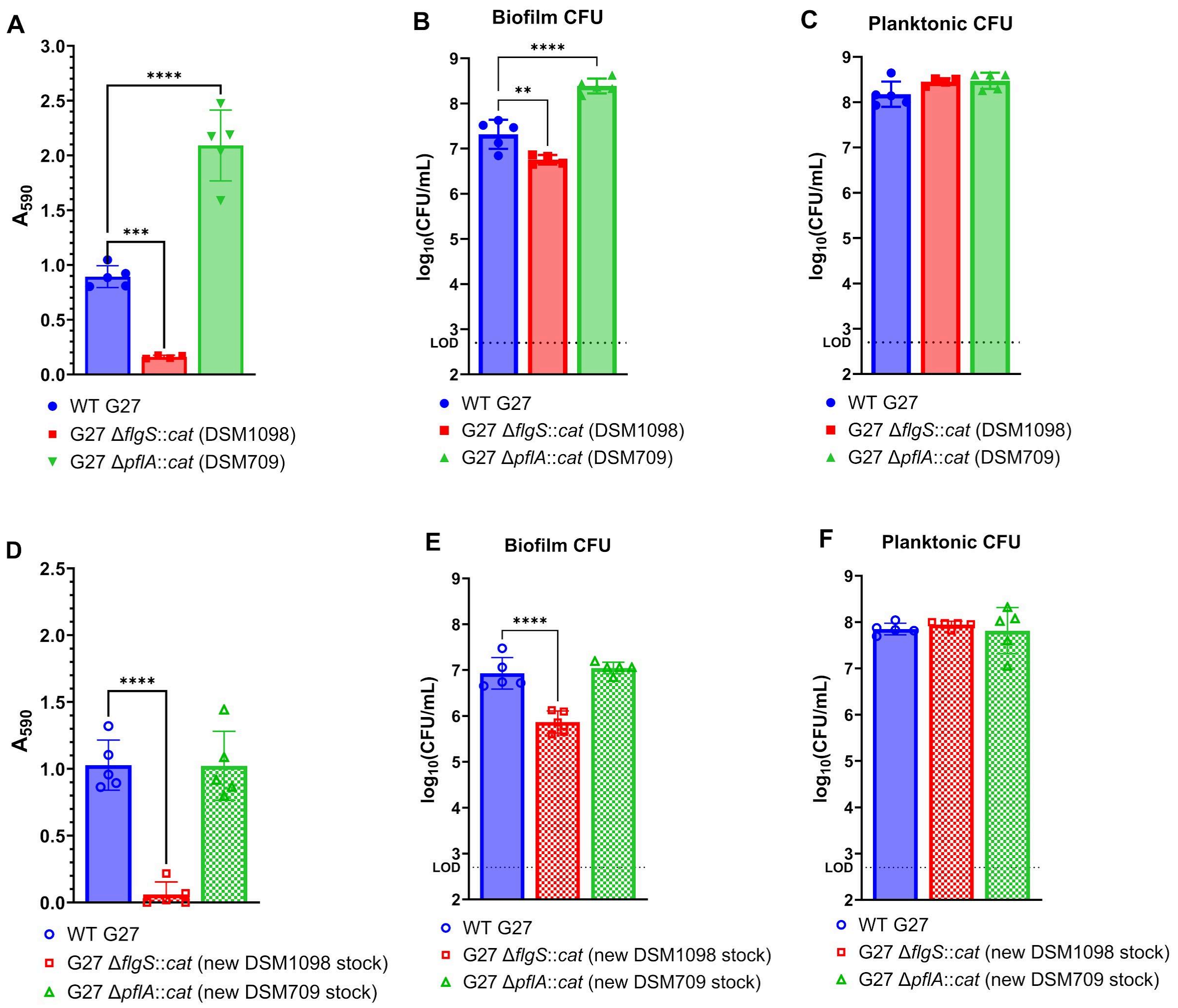

Figure S1. At 72-hours, the original G27 *flgS* and *pflA* deletion insertion mutant strains displayed significant biofilm deficient and hyper-biofilm phenotypes, respectively, but the phenotype of G27 Δ*pflA*::*cat* was lost after laboratory expansion. (A and D) OD-controlled overnight cultures were adjusted to an OD_{600} of 0.1 and added to a 24-well plate. After 72 hours, the biofilms were washed with BB, allowed to dry, and then stained with 1% crystal violet. Stained biomass was resuspended in an alcohol solution and the absorbance was read at 590 nm. (B-C and E-F) A duplicate set of wells was used to collect planktonic and biofilm CFU. (A-C) Original stocks of the G27 Δ*flgS*::*cat* (DSM1098) and Δ*pflA*::*cat* (DSM709) mutant strains were tested in the biofilm assay. (D-F) Over a year later, after expansion of both DSM1098 and DSM709 to create new freezer stocks, G27 Δ*flgS*::*cat* and Δ*pflA*::*cat* mutant strains were retested in the biofilm assay. Both stocks were expanded under antibiotic selection to maintain the chloramphenicol cassette (*cat*); PCR on gDNA preps from the new stocks was used to confirm that the original

gene deletions were still present. However, G27 Δ*pflA*::*cat* lost the original hyper-biofilm phenotype while G27 Δ*flgS*::*cat* was still biofilm deficient. One-way ANOVAs with Dunnett correction were performed in comparison to WT G27; CFU were log-transformed for analysis; limit of detection (LOD, dotted line) was 500 CFU; n≥4; individual data points are plotted with mean and SD; **** P<0.0001, *** P=0.0004, and ** P=0.0063 for the indicated comparisons.

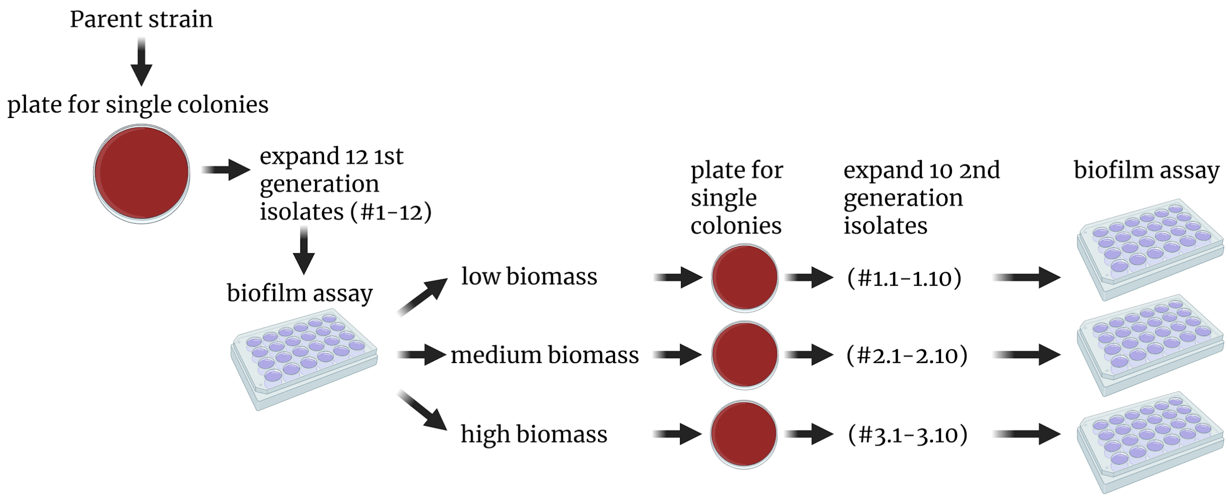

Figure S2. Overview of the single colony isolation and screening approach. These steps were repeated for each parent strain, DSM1 (lab WT G27), DSM359 (low-passage G27), and DSM136 (SS1). Created in BioRender (available from https://BioRender.com/eanb1o3).

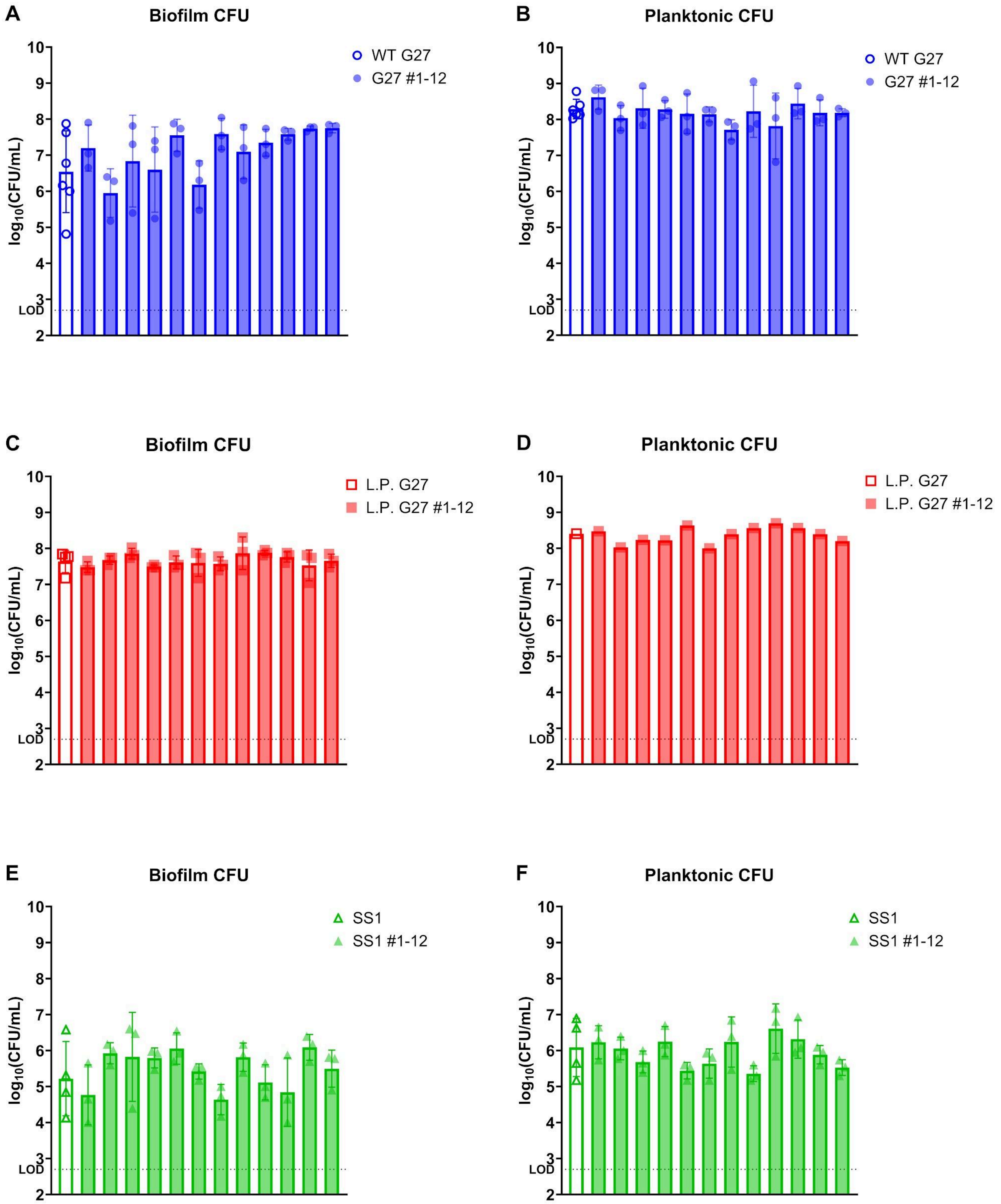

Figure S3. Biofilm and planktonic CFU data for all first-generation single colony isolates. (A, C, and E) Biofilm and (B, D, and F) planktonic CFU data from WT G27, L.P. G27, and SS1 first-generation single colony isolates, respectively; data are from the same biomass experiments depicted in Figure 2. Biofilms of SS1 isolates were grown in BB+1% FBS, while WT

G27 and L.P. G27 isolates were grown in BB10. One-way ANOVAs with Dunnett correction were performed on log-transformed data in comparison to the parent strain (open bar on each graph); limit of detection (LOD, dotted line) was 500 CFU; $n \geq 3$; individual data points are plotted with mean and SD; no significant differences were detected.

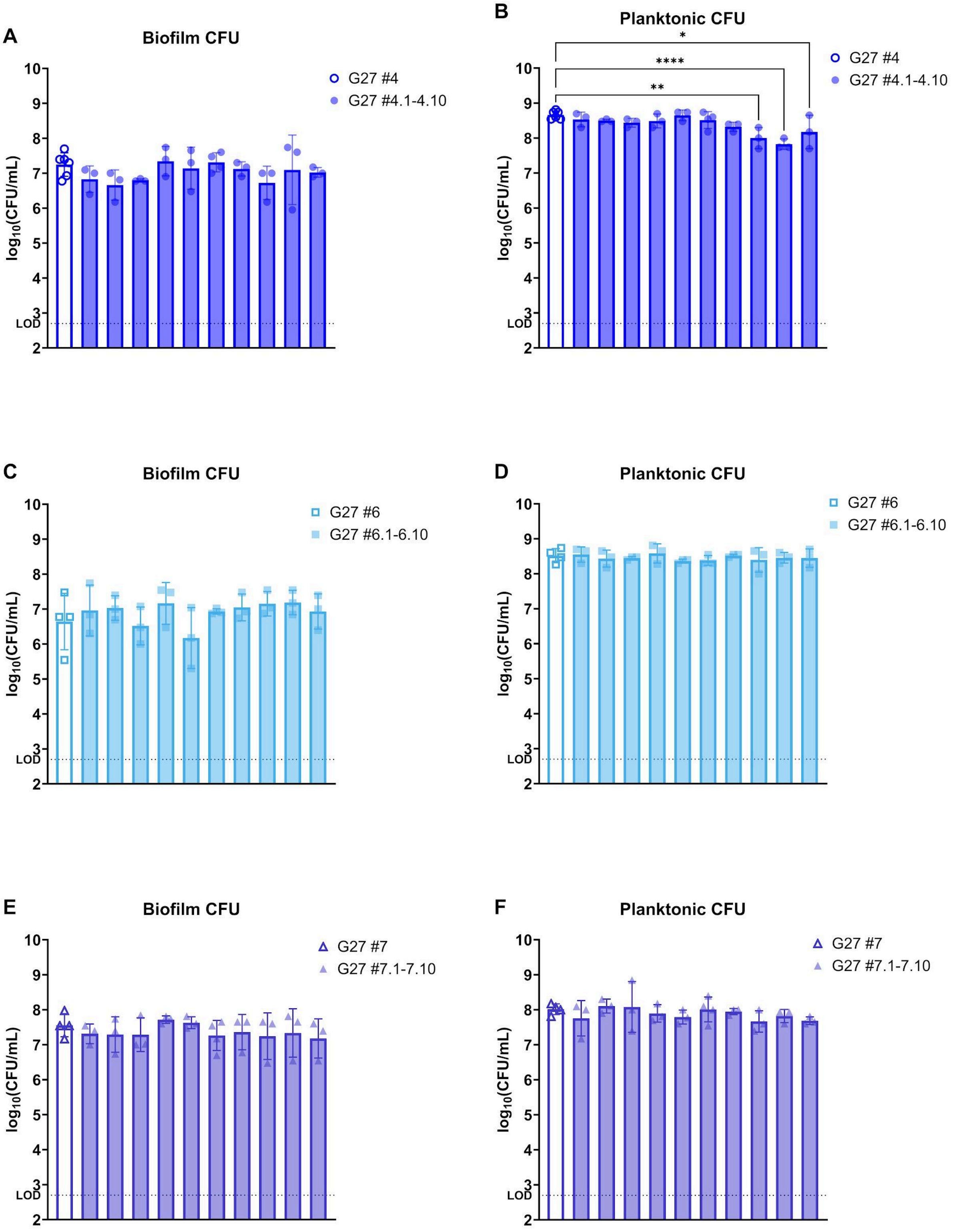

Figure S4. Biofilm and planktonic CFU data for WT G27 second-generation single colony isolates. (A, C, and E) Biofilm and (B, D, and F) planktonic CFU data from G27 #4.1-4.10,

#6.1-6.10, and #7.1-7.10, second-generation single colony isolates, respectively; data are from the same biomass experiments depicted in Figure 3A. One-way ANOVAs with Dunnett correction were performed on log-transformed data in comparison to the parent strain (open bar on each graph); limit of detection (LOD, dotted line) was 500 CFU; $n \geq 3$; individual data points are plotted with mean and SD; **** $P<0.0001$, ** $P=0.0016$, and * $P=0.0296$ for the indicated comparisons.

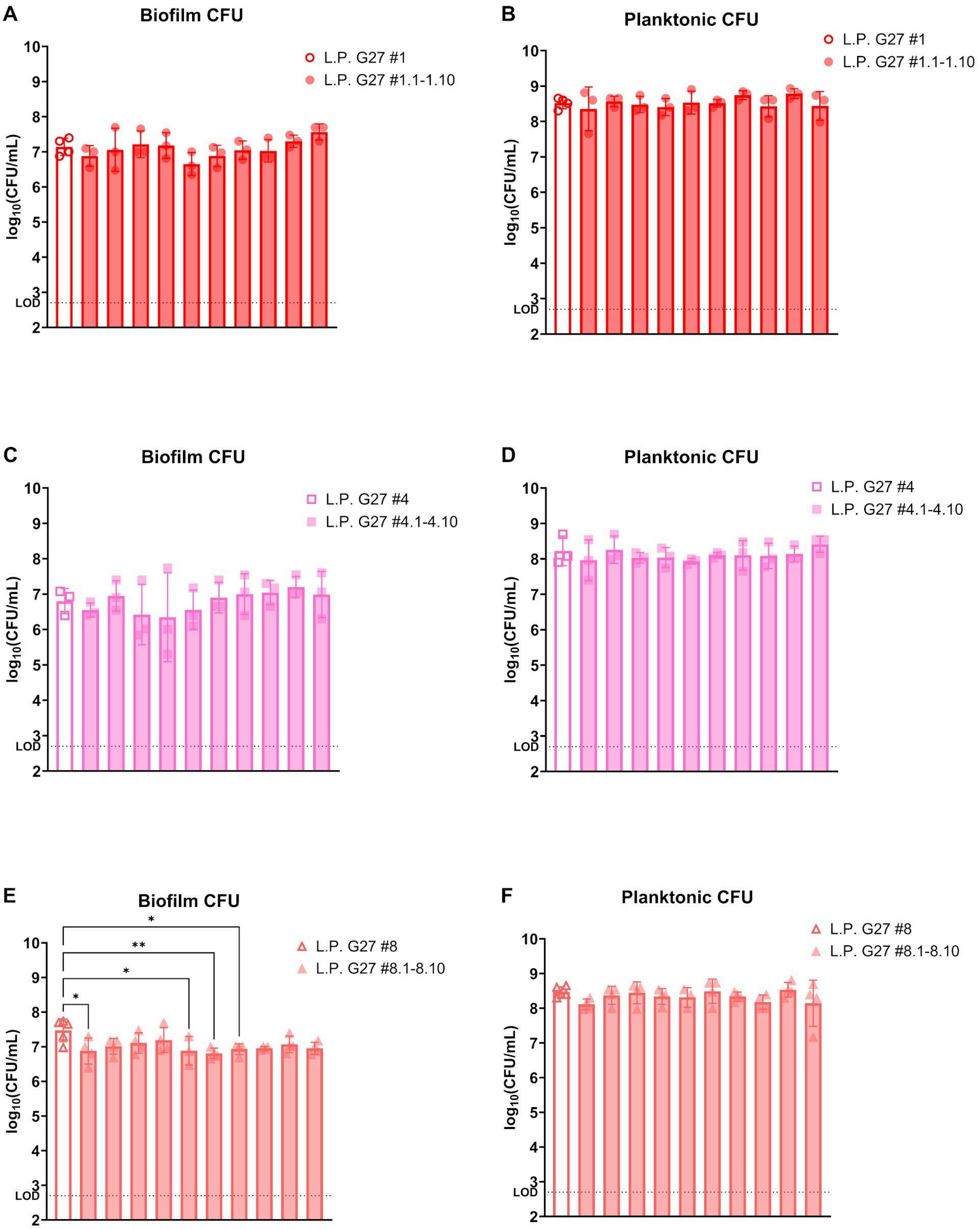

Figure S5. Biofilm and planktonic CFU data for L.P. G27 second-generation single colony isolates. (A, C, and E) Biofilm and (B, D, and F) planktonic CFU data from L.P. #1.1-1.10, #4.1-4.10, and #8.1-8.10, second-generation single colony isolates, respectively; data are from

the same experiments depicted in Figure 3B. One-way ANOVAs with Dunnett correction were performed on log-transformed data in comparison to the parent strain (open bar on each graph); limit of detection (LOD, dotted line) was 500 CFU; $n \geq 3$; individual data points are plotted with mean and SD; ** $P=0.0075$ and * $P \leq 0.04$ for the indicated comparisons.

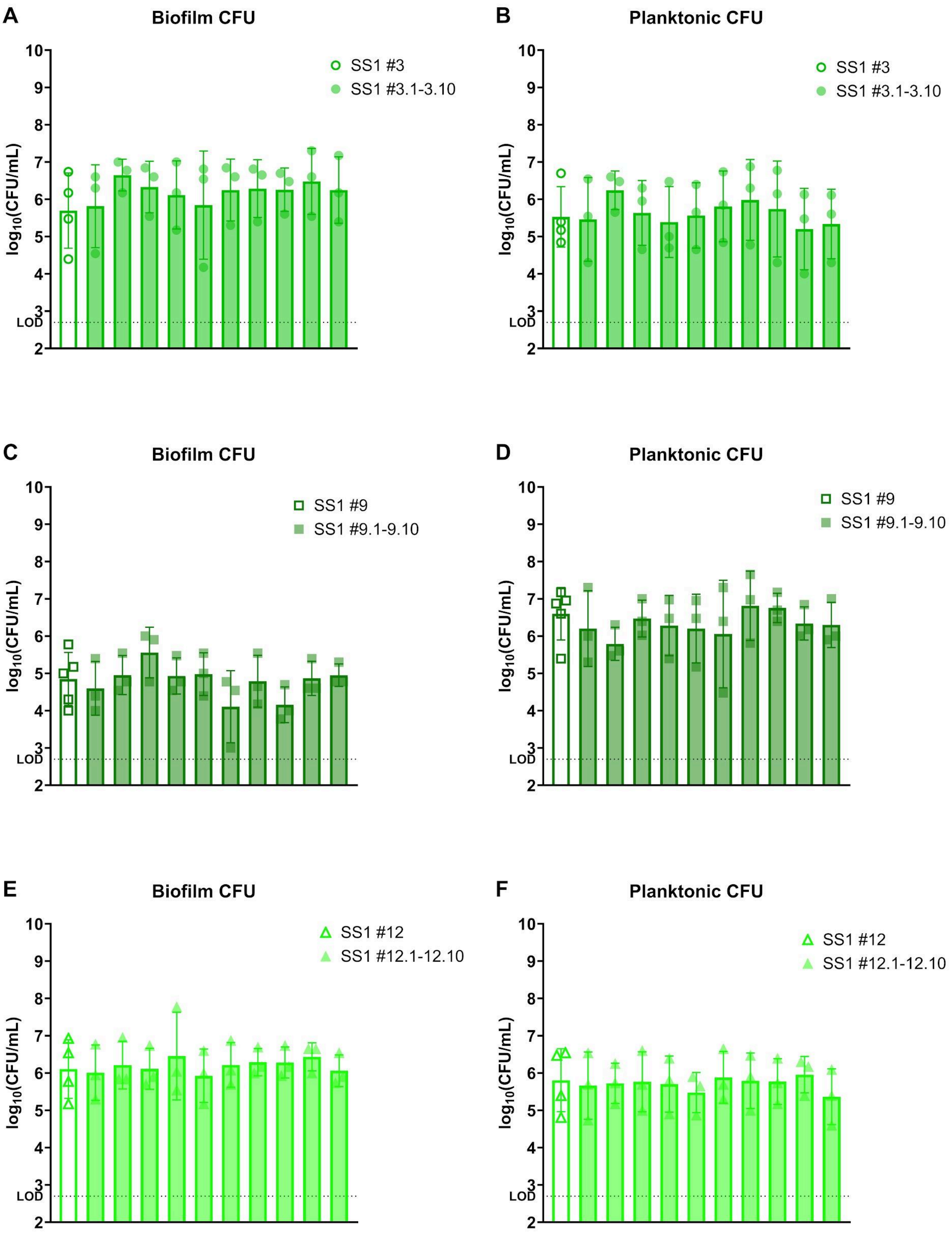

Figure S6. Biofilm and planktonic CFU data for SS1 second-generation single colony isolates. (A, C, and E) Biofilm and (B, D, and F) planktonic CFU data from SS1 #3 and #3.1-3.10, #9 and

#9.1-9.10, and #12 and #12.1-12.10, respectively; data are from the same biomass experiments depicted in Figure 3C. Biofilms were grown in BB+1% FBS. One-way ANOVAs with Dunnett correction were performed on log-transformed data in comparison to the parent strain (open bar on each graph); limit of detection (LOD, dotted line) was 500 CFU; $n \geq 3$; individual data points are plotted with mean and SD; no significant differences were detected.

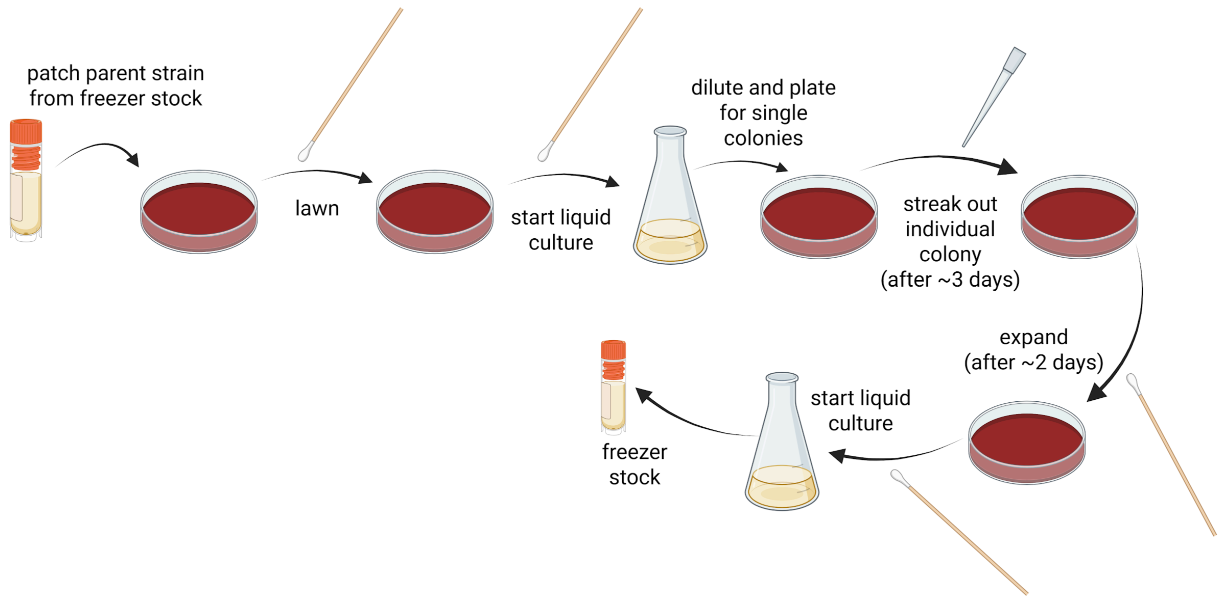

Figure S7. Growth and expansion details for *H. pylori* single colony plating and isolation. This protocol was used for all parent strains (DSM1, DSM359, DSM136, and first-generation isolates selected for second-generation colony isolation) to represent normal *in vitro* passage methods for *H. pylori*. Unless otherwise noted, each step occurred after ~18-24 hours. The freezer stock for each new single colony isolate was used for downstream assays such as biofilm formation, motility, and gDNA isolation for WGS and variant calling. Created in BioRender (available from https://BioRender.com/tuirv7n).

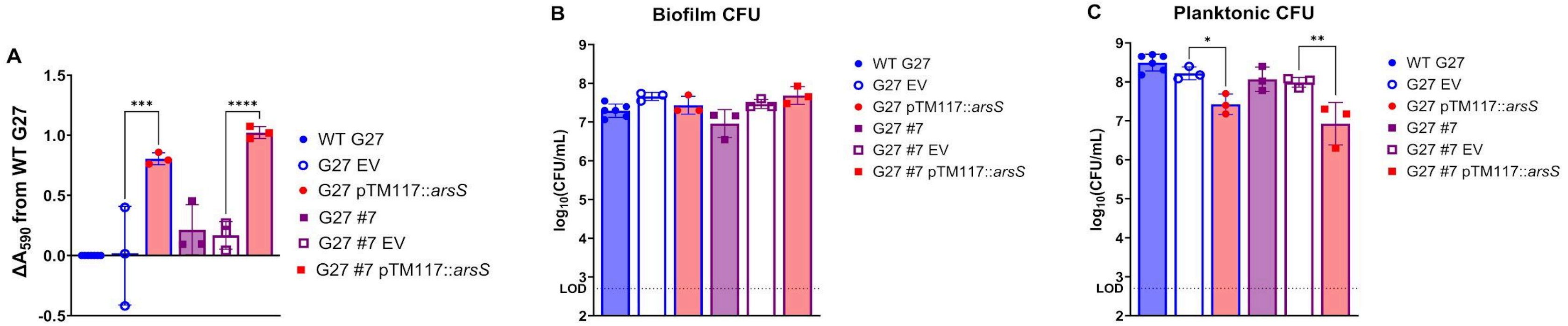

Figure S8. Adding a WT copy of *arsS* on an expression plasmid (pTM117::*arsS*) to WT G27 (DSM1) or first-generation single colony isolate G27 #7 results in increased biomass as compared to the respective empty vector (EV) control strains. (A) OD-controlled overnight cultures were adjusted to an OD_{600} of 0.1 and added to a 24-well plate. After 72 hours, the biofilms were washed with BB, allowed to dry, and then stained with 1% crystal violet. Stained biomass was resuspended in an alcohol solution and the absorbance was read at 590 nm. (B-C) A duplicate set of wells was used to collect planktonic and biofilm CFU; limit of detection (LOD, dotted line) was 500 CFU. One-way ANOVAs with Bonferroni correction were performed on baseline-corrected data (A) or on log-transformed CFU data (B-C); n≥3; individual data points are plotted with mean and SD; **** P<0.0001, *** P=0.0002, ** P=0.0028, and * P=0.0291 for the indicated comparisons. Seven preplanned comparisons of interest were performed; the following biomass comparisons were not significant: WT G27 vs G27 EV, WT G27 vs G27 #7, G27 #7 vs G27 #7 EV, G27 EV vs G27 #7 EV, and G27 pTM117::*arsS* vs G27 #7 pTM117::*arsS*; only two of the seven preplanned comparisons were significant in the planktonic CFU dataset, as indicated.

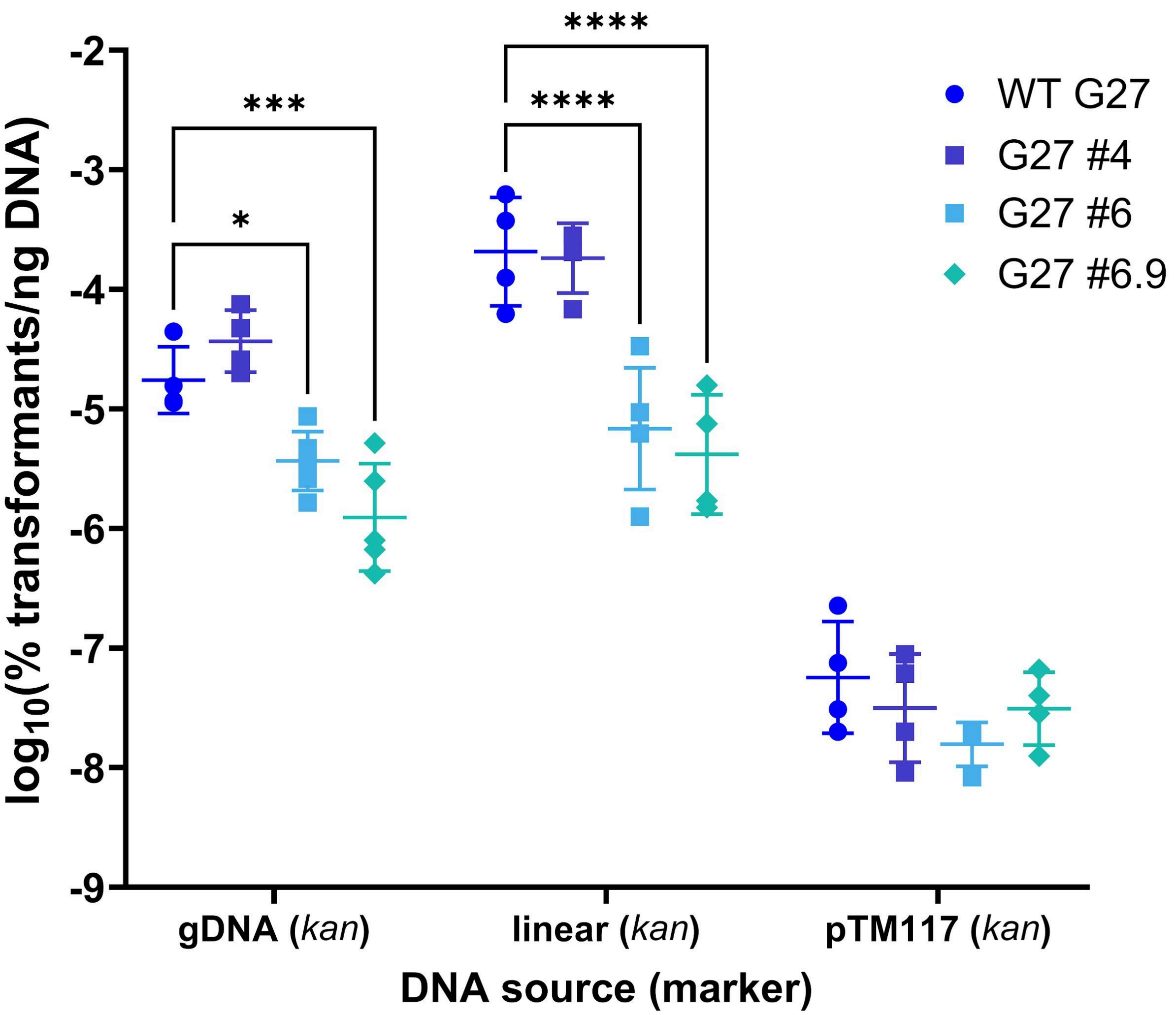

Figure S9. First-generation single colony isolate G27 #6 displays a transformation efficiency defect that is maintained in the tested second-generation isolate, G27 #6.9. Transformation efficiency (% transformants) was calculated by diluting, spot plating, and counting CFU on selective (HBA+Kan25) and non-selective (HBA) plates after each transformation; limit of detection was 500 CFU. Percent transformants was then divided by the amount of DNA (ng) used to transform each sample; 500 ng of DNA per transformation was used for plasmid and gDNA samples and 250 ng was used in each transformation of linear DNA (~2kb PCR product). A two-way ANOVA with Dunnett correction was performed on log-transformed data in comparison to WT G27; n≥4; individual data points are plotted with mean and SD; * P<0.03, *** P=0.0002, and **** P<0.0001 for the indicated comparisons.
